# Population Thinking and the Uniqueness of Biological Entities

**DOI:** 10.1007/s10441-025-09498-0

**Published:** 2025-06-13

**Authors:** Daniel J. Nicholson

**Affiliations:** Department of Philosophy, George Mason University, 4400 University Drive, Fairfax, VA 22030 USA

**Keywords:** Population thinking, Uniqueness, Variation, Non-genetic heterogeneity, Ernst Mayr

## Abstract

The concept of ‘population thinking’ was introduced by Ernst Mayr in the mid-twentieth century and it has since become one of the most pervasive notions in the philosophy of biology. Despite its influence, however, the term has been widely misunderstood, even by those who have done the most to champion it. Population thinking today is often confused with population-level thinking (i.e., the idea of treating populations as units of analysis), which, ironically, is the opposite of what Mayr intended to convey when he coined the term. For Mayr, population thinking was a way of emphasizing the variation among individuals in a population, as well as the importance of recognizing their differences and uniqueness. In this paper, I recover the original meaning of ‘population thinking’ and elucidate its central role in evolutionary theory. I also demonstrate its surprising relevance to many other areas of contemporary biology. In particular, I show how the recent introduction of novel methodologies in molecular biology has led to a number of unexpected discoveries that are best understood through the lens of population thinking. Finally, I examine the historical origins and philosophical foundations of population thinking, and I show how it lies at the heart of what makes biology different from physics.

## Introduction

The idea of ‘population thinking’, introduced by Ernst Mayr in the mid-twentieth century, is one of the most well-known and frequently discussed notions in the philosophy of biology. The *locus classicus* is a short essay that Mayr penned on the occasion of the centennial of the *Origin of Species* (Mayr 1959a), which later appeared in abridged form in a widely read collection of his key writings (Mayr 1976: 26–29).^1^ Mayr identifies population thinking as one of Charles Darwin’s main achievements, alongside the evidence he marshalled in support of evolution, and his principle of natural selection. Darwin’s population thinking supplanted a longstanding mode of thinking that Mayr calls ‘typological thinking’. This refers to the idealist belief, ostensibly dating back to Plato, that the bewildering diversity found in nature can be neatly classified into a limited number of fixed classes or pre-existing ‘types’, which underlie the observed variability. In a famous, oft-quoted passage, Mayr states that

The assumptions of population thinking are diametrically opposed to those of the typologist. The populationist stresses the uniqueness of everything in the organic world. What is true for the human species—that no two individuals are alike—is equally true for all other species of animals and plants. Indeed, even the same individual changes continuously throughout its lifetime and when placed into different environments. All organisms and organic phenomena are composed of unique features and can be described collectively only in statistical terms. Individuals, or any kind of organic entities, form populations of which we can determine only the arithmetic mean and the statistics of variation. Averages are merely statistical abstractions; only the individuals of which the populations are composed have reality. The ultimate conclusions of the population thinker and of the typologist are precisely the opposite. For the typologist, the type (*eidos*) is real and the variation an illusion, while for the populationist the type (average) is an abstraction and only the variation is real. No two ways of looking at nature could be more different. (Mayr 1959a: 2)

In *Animal Species and Evolution*, and in many of his publications thereafter, Mayr proclaims that “[t]he replacement of typological thinking by population thinking is perhaps the greatest conceptual revolution that has taken place in biology” (Mayr 1963: 5–6). In fact, as Carl Chung (2003) has shown, the typological/population distinction is a central conceptual thread that runs throughout Mayr’s scientific, historical, and philosophical work. He uses it (a) to account for the crucial innovation in Darwin’s theory (e.g., Mayr 1977, 1991, 2001), (b) to explain the origin and development of his biological species concept (e.g., Mayr 1963, 1968, 1969), (c) to support his interpretation of the Modern Synthesis and justify his critiques of the early Mendelians and of theoretical population genetics (e.g., Mayr 1959b, 1982; Mayr and Provine 1980), (d) to ground his attack on racism (e.g., Mayr 1959a, 1976, 1997), and (e) to defend the autonomy of biology (e.g., Mayr 1985, 1988, 2004)—a topic I shall turn to at the end of the paper. Mayr’s protégé Walter Bock (1994: 285) remarked that “[n]othing in Mayr’s most general theoretical writings has been more important than his stand against typology in biology”, which stemmed from his “unwavering belief in ‘population thinking’” (ibid.).^2^

Open any philosophy of biology textbook today and you will find at least a few pages devoted to population thinking (e.g., Sterelny and Griffiths 1999: 7–10; Godfrey-Smith 2014: 139–143; Okasha 2019: 17–19). The same is true for textbooks on the history of biology (e.g., Sapp 2003: 28–29; Bowler 2003: 145, 156, 162–163) and even evolutionary biology itself (e.g., Ridley 2004: 363–365). And that is not all. Population thinking is discussed in wider contexts still, such as in evolutionary economics (e.g., Metcalf 1998; Hodgson 2002; Andersen 2004) and most recently in cultural evolution (e.g., Houkes 2012; Lewens 2015; Baravalle 2021).^3^

In spite of its influence, or perhaps because of it, the typological/population distinction has recently been challenged on various fronts (e.g., Winsor 2003, 2006; Amundson 2005; Levit and Meister 2006; Wilkins 2009; Müller-Wille 2011; Witteveen 2015, 2016). The rather damning verdict of historians of biology is that Mayr erected a typological strawman, which had the effect of conflating existing distinctions and obscuring previously meaningful contrasts—all in the service of attacking an indefensible caricature. He also problematically equated typological thinking with *essentialism*, muddling both doctrines even further, and then projected these fraught labels back into history, making sweeping generalizations in the process. In addition, Mayr legitimized his idea of population thinking by anachronistically tying it to Darwin (who never used that term), thereby exaggerating the gap between pre-Darwinian and post-Darwinian biology. Finally, he strategically employed the typological/population distinction to opportunistically intervene in a number of unrelated theoretical debates, resulting in even more confusion.

It is undeniable that Mayr was often less than rigorous in his use of the typological/population distinction. For instance, in the famous 1959 essay quoted above, after asserting that “gradual evolution is basically a logical impossibility for the typologist” (Mayr 1959a: 2), he notes that “[v]irtually every controversy in the field of evolutionary biology […] was a controversy between a typologist and a populationist” (ibid.: 2–3). Further down the same page, Mayr attributes to the typologist six theses about natural selection, all in the space of a single paragraph. Specifically, he claims that the typologist is committed to the following: (i) everything in nature is either ‘good’ or ‘bad’, (ii) natural selection is an all-or-nothing phenomenon, (iii) evolution consists in the testing of newly arisen ‘types’, (iv) evolution is aptly characterized as ‘survival of the fittest’, (v) natural selection does not work, and (vi) other forces are responsible for evolution. Not only can these theses be held independently (i.e., one can accept some and reject others), but it is not even clear that they can all be held consistently. Mayr discusses many other examples of typological thinking elsewhere in his writings. ‘The typologist’ (and later, ‘the essentialist’) is a moving target for Mayr, capable of adopting different forms depending on the context.

There is certainly much that is objectionable in the way Mayr exploited the typological/population distinction in different areas of his work. However, my impression is that his ideas have been overshadowed by the efforts of later scholars to prove him wrong. Without wanting to call for a full-scale rehabilitation of Mayr’s views (after all, criticizing Mayr is almost a rite of passage for historians and philosophers of biology), I do think that recent scholarship has come dangerously close to throwing the baby out with the bathwater. There are, I believe, fascinating yet largely unexplored aspects of Mayr’s treatment of population thinking that can be extremely helpful to think about in the light of contemporary biology.

Despite the vast body of literature that the typological/population distinction has generated, I want to suggest that we have lost sight of Mayr’s original insights. The most dramatic illustration of this is that today the very concept of population thinking is often construed to mean almost the opposite of what Mayr meant to convey when he proposed it. Population thinking nowadays is usually invoked in evolutionary contexts—particularly in discussions of population genetics—to emphasize the importance of focusing on the properties of populations instead of on the properties of the individuals that constitute them. Paradoxically, this is the inverse of what Mayr sought to highlight, which were the crucial, irreducible differences that exist between individuals in any biological population.

My aim in this paper is to reclaim what I think is most valuable in the idea of population thinking, namely its recognition of the ontological uniqueness of biological entities, and to show its importance in a variety of biological contexts. My analysis differs substantially from recent practice-oriented reappraisals of Mayr’s typological/population distinction (Brigandt 2007; Lewens 2009; Love 2009; DiTeresi 2010; Witteveen 2018; Suzuki 2021) in that it returns the focus to the metaphysical issues, which were Mayr’s original concern. I proceed as follows. After surveying and sorting the sprawling literature on population thinking, typology, and essentialism (Sect. 2), I argue that population thinking has become deeply entangled with a very different viewpoint, which I call ‘population-level thinking’ (Sect. 3). I then examine the theoretical significance of population thinking (Sect. 4), and I show how it can help us make sense of surprising empirical findings in contemporary molecular biology (Sect. 5). Finally, I reflect on the nature of biological uniqueness and on the bearing of population thinking on the relation between biology and physics (Sect. 6).

## Population Thinking, Typology, and Essentialism

The provocative contrast that Mayr drew between population thinking on the one hand and typological thinking and essentialism on the other has received a great deal of attention in recent years. Many historians have flatly rejected the historical narrative that Mayr offered to justify his distinction, denouncing it as a pernicious myth that Mayr mischievously fabricated. Polly Winsor (2003, 2006) irreverently dubbed it the ‘Essentialism Story’, and the term has stuck. It goes something like this: before Darwin, naturalists interpreted the differing organisms in a species as imperfect manifestations of a Platonic type or ideal. Species were held to be classes, or natural kinds, defined by a fixed set of essential characters, thereby rendering the idea of evolution inconceivable. Darwin overcame this essentialism by adopting a sort of nominalism according to which every member of a species, and every species, is a unique particular. In place of the traditional metaphysics of essentialism, Darwin reconceptualized species as variable populations evolving over time.

As it turns out, there is surprisingly little historical evidence to substantiate the Essentialism Story. Species fixism is not an ancient doctrine (Wilkins 2009); it only acquired currency in the mid-eighteenth century—about a hundred years before Darwin—and even then, it was not grounded in essentialist metaphysics but was rather an empirical hypothesis that was arrived at inductively, as in the case of the Linnaean system of classification (Müller-Wille 2011). The typological thinking of the idealistic morphologists of the nineteenth century was not essentialist either. Types were not defined in terms of necessary and sufficient conditions, and in any event species were seldom types themselves, but more often representatives of types (Amundson 2005).^4^

When typological views experienced a resurgence among German morphologists and paleontologists in the first half of the twentieth century, the notion of type that was invoked referred to an abstract pattern of organization devised for the purpose of comparing and classifying body plans. Typological analyses of this kind were non-causal and made no claims about phylogenetic history, which means that they were not in conflict (at least not in principle) with Darwinian explanations. They were also not Platonic or essentialist. The postulated types were not even necessarily invariant; in fact, they were often construed dynamically (Levit and Meister 2006).

Reviewing the history of biology, one is hard pressed to find a single instance of a typological essentialist *in Mayr’s precise sense*. Aristotle wasn’t, and neither was Carl Linnaeus. Not Johann Wolfgang von Goethe, not Étienne Geoffroy Saint-Hilaire, and not Richard Owen. Louis Agassiz probably comes the closest, and Mayr did publish an extensive study of Agassiz the same year he fully articulated his typological/population distinction (Mayr 1959c), but Agassiz’s typological views are quite idiosyncratic.^5^ The fact is that the notion of ‘type’ has lent itself to numerous interpretations throughout history: some metaphysical and theoretical, others practical and instrumental. A primary motivation for exposing the Essentialism Story has been to disassociate typology from essentialism.^6^ By intertwining typology with essentialism, Mayr obscured the various legitimate roles that typological thinking, in its various incarnations, has played in the history of taxonomy. For example, William Whewell’s ‘Method of Type’, which construed types not as idealized abstractions ranging over a group but as concrete exemplars of a group, was an important milestone in the development of systematics.

**Fig. 1:**
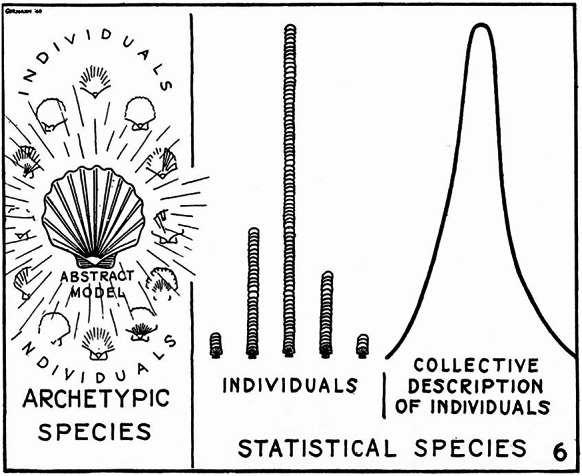
Simpson’s illustration—18 years before Mayr’s famous paper—of the contrast between ‘archetypic’ and ‘population-statistical’ approaches to species taxonomy (after Simpson 1941)

Recent scholarship has also revealed that Mayr came up with his typological/population distinction by fusing together several distinctions previously developed in different contexts by two of his close friends and colleagues, namely George Gaylord Simpson and Theodosius Dobzhansky. Joeri Witteveen (2015) has carefully documented how from 1937 onwards both Simpson and Dobzhansky started contrasting distinct notions of ‘type’ in their work with what they each characterized as ‘populational’ approaches. Simpson took issue with the methodological use of individual type specimens as definite standards governing the construction of classifications in paleontological taxonomy, advocating instead for a statistically based ‘population systematics’. Simpson (1941: 12) called for a shift from “the old, static, pseudo-archetypal taxonomy” towards “the new, dynamic, statistical taxonomy”, illustrating this with a diagram reproduced in Fig. 1. Dobzhansky, for his part, criticized the appeal to the ‘type’ concept in biological discussions of race, where it was insidiously construed as “a sort of noumenon of which existing individuals are only imperfect representatives” (Dobzhansky 1951: 264), defending in its place a view of races as somewhat arbitrary demarcations of genetically variable populations. Dobzhansky also drew a type/population contrast in his much publicized ‘classical/balance’ controversy with Hermann Joseph Muller regarding the existence of optimal homozygous ‘wild-types’, the genetic constitution of natural populations, and the operation of natural selection (see Chung 2000).

Witteveen (2016) has also shown that in *Systematics and the Origin of Species* (Mayr 1942), Mayr began to weave Simpson’s methodological criticisms of type specimens as classificatory standards into his own theoretical critique of the traditional morphological species concept, and he drew on the new populational approach to systematics to formulate his biological species concept. A decade later, Mayr began to explicitly refer to the morphological species concept as ‘typological’, and he also started associating the idea of types with Plato’s metaphysics (Chung 2003). In 1955 he went a step further, broadening the scope of the typological/population distinction from taxonomy to biology as a whole (Mayr 1955). By the time of his famous 1959 paper on the distinction (Mayr 1959a), he had also subsumed Dobzhansky’s earlier contrasts between typological and populational conceptions of race and of natural selection. Mayr would use the distinction for yet another purpose in 1959, namely, to critically evaluate the contributions of population genetics to the Modern Synthesis (Mayr 1959b). In his later writings, Mayr continued to invoke the distinction in even further contexts.

Interestingly, while the response to Mayr’s *historical* claims about the typological/population distinction—especially in recent years—has been mostly negative (but see Stamos 2005), the reception of his *philosophical* claims against essentialism, and in support of population thinking, has been much more positive. Mayr’s frequent, full-throated attacks on species essentialism roughly coincided with the championing of essentialist views about natural kinds by prominent analytic philosophers like Hilary Putnam (1975) and Saul Kripke (1980), who argued that the aim of science is to discover these essentialist kinds. Chemistry appears to provide a striking vindication of essentialism: the periodic table of elements is quite literally a taxonomy of chemical natural kinds, each of which is defined by an intrinsic essential property—the atomic number—possessed by all and only those members of that kind. Nevertheless, Putnam’s and Kripke’s essentialism was intended to apply to *all* sciences, including biology. Mayr’s views, together with those of Hull (1965), eventually hardened into a robust anti-essentialist consensus among philosophers of biology (e.g., Sober 1980; Dupré 1981). By the time Sober wrote his textbook *Philosophy of Biology* in 1993, he could justifiably declare that “essentialism about species is today a dead issue” (Sober 1993: 163). Even so, many have found it difficult to give up the intuitive idea that species taxa pick out something real in the living world.

Accordingly, we can interpret the recent literature on this topic as different attempts to resolve what Matt Haber (2016) fittingly describes as an ‘inconsistent triad’ of theses regarding the nature of species, namely:

Thesis 1: *Species are paradigmatic natural kinds*.

Thesis 2: *Natural kinds are defined by essential properties*.

Thesis 3: *Species do not have essential properties*.

Michael Ghiselin (1974) and Hull (1976) famously rejected the first thesis, arguing that species are not kinds but *individuals*—concrete particulars bound in space and time (I will return to this proposal later in the paper). Some have opted instead to modify the second thesis by reconceptualizing the essential properties that determine kind membership *extrinsically* instead of intrinsically. Paul Griffiths (1999), Samir Okasha (2002), and Joseph LaPorte (2004) all argue that species members share ‘historical’ or ‘relational’ essential properties, such as deriving from a common ancestor, belonging to a certain interbreeding population, or occupying the same ecological niche. Others have simply denied the second thesis altogether. Richard Boyd (1999) proposed to replace the strict definition of natural kinds in terms of necessary and sufficient essential properties in favor of a more flexible characterization of them as *homeostatic property clusters*—a suggestion that has been developed by Rob Wilson (1999) and Olivier Rieppel (2013), and in slightly revised form by Matthew Slater (2015). Then there are those who have taken issue with the third thesis. Denis Walsh (2006) and Christopher Austin (2019) have both claimed that even if there are no essences about particular species taxa, organisms still possess causally efficacious essential properties by virtue of their robust developmental ‘natures’, which are determined by gene regulatory networks and other supraspecific developmental modules. Finally, Michael Devitt (2008, 2023) and Stephen Boulter (2012) have gone as far as to resurrect full-blown Aristotelian essentialism about species. Table 1 summarizes this remarkable range of responses.

**Table 1:** Recent contributions to the philosophical literature on essentialism in biology, interpreted as different attempts to resolve an inconsistent triad of theses regarding the metaphysics of species

Thesis	Position	Proposal	Key References
Species are paradigmatic natural kinds	Rejection	Species-as-individuals thesis	Ghiselin 1974; Hull 1976
Natural kinds are defined by essential properties	Revision	Historical or relational essentialism	Griffiths 1999; Okasha 2002; LaPorte 2004
	Rejection	Homeostatic property cluster theory	Boyd 1999; Wilson 1999; Rieppel 2013; Slater 2015
Species do not have essential properties	Revision	Non-taxonomic essentialism	Walsh 2006; Austin 2019
	Rejection	Intrinsic biological essentialism	Devitt 2008, 2023; Boulter 2012﻿

While some have sought to decouple essentialism from typology in the hope of rehabilitating the former, others have done so with the intention of rehabilitating the latter—arguing for the contemporary relevance of typological thinking in areas like evolutionary developmental biology, where talk of morphological and developmental types is almost inevitable. Ron Amundson (1998) was the first to do so, and many others have followed in the years since (Brigandt 2007; Lewens 2009; Love 2009; DiTeresi 2010; Witteveen 2018). These reappraisals of typological thinking are characterized by a desire to redirect our attention toward considerations of scientific practice and to leave behind the metaphysical issues, which are perceived to be irrelevant at best and ill-conceived or meaningless at worst.

Overall, the most striking thing about the amount of ink that historians and philosophers of biology have spilled on the topic of population thinking over the years is how extraordinarily little attention the very idea of population thinking has received. The focus is invariably on what population thinking is a reaction against or is supposed to have historically overthrown—be it typology or essentialism. Population thinking itself, for the most part, is simply taken for granted as a core tenet of modern biology, and as “the *right* way of thinking about the biological domain” (Nanay 2010: 92). Bence Nanay is not wrong when he observes that “Ernst Mayr’s concept of population thinking […] must be the most often invoked, yet, most unexplained in the philosophy of biology” (ibid.).

The very few authors who have carefully examined Mayr’s population thinking on its own terms have typically reclaimed it for other purposes—and in appropriating the concept to advance their own agenda (instead of Mayr’s), they have fundamentally changed its meaning. André Ariew has done this in a chapter for *The Oxford Handbook of Philosophy of Biology* titled ‘Population Thinking’ (Ariew 2008). After criticizing Mayr for embroiling population thinking in abstruse metaphysical questions concerning the reality of types and individuals, Ariew proceeds to completely redefine the concept so that it refers to the epistemic strategy of explaining large-scale population-level events (such as extinction, speciation, and adaptation) as the aggregative result of the activities of large numbers of individuals. A not-so-hidden motivation for Ariew’s reconceptualization of population thinking is to vindicate—historically as well as philosophically—his own statisticalist interpretation of natural selection (see Walsh et al. 2017).

Sober did the very same thing (albeit a bit more subtly) many years earlier in a widely read and rightfully celebrated paper titled ‘Evolution, Population Thinking, and Essentialism’ (Sober 1980). This paper is credited with providing an alternative account of the essentialist or typological tradition (Sober, like Mayr, does not distinguish the two) that Darwinian population thinking supposedly did away with. Essentialism for Sober involves a commitment to what he calls the *natural state model*, which he traces back to Aristotle. Rather than ignoring variation (as Mayr claimed), essentialists explain variation as deviations from the natural tendency of an entity—be it an organism, a population, or anything else—to realize its ‘natural state’.^7^ What has not been generally recognized, however, is that Sober’s paper also offered an alternative account of population thinking itself, which he construed as the sort of thinking found in population biology. Sober’s reconceptualization of population thinking, like Ariew’s, was driven by his own agenda at the time, which included justifying the explanatory successes of population genetics and interjecting in the then-raging units of selection debate (see Sober and Lewontin 1982).^8^

As I will argue in the next section, Sober’s appropriation of Mayr’s population thinking (and to a lesser extent, Ariew’s) is responsible for considerable confusion regarding the way this term is used and understood today, and it has inadvertently led us to neglect what Mayr intended to convey by coining it.

## Population Thinking ≠ Population-Level Thinking

So, what exactly *is* population thinking according to Mayr? Given how fickle and opportunistic he is supposed to have been in his deployment of the typological/population distinction over the course of his extraordinarily long career, one may harbor serious doubts that this question can be answered at all. Perhaps it is futile to try to reconstruct what Mayr *really* meant by population thinking because he never tethered the concept to a single definition to begin with, but instead co-opted and reformulated it in different ways depending on the situation he found himself in. In this vein, some scholars have been inclined to conclude that “the very distinction between typological thinking and population thinking is a piece of mere rhetoric that was concocted and rehearsed for purely strategic, programmatic reasons” (Witteveen 2018: 123) and just leave it at that.

I do not think this is quite right. Although the target of Mayr’s criticisms did change from one context to another (and with it his negative account of typological thinking and essentialism), his basic positive conception of population thinking did not. In fact, when we survey Mayr’s extensive oeuvre we find him being surprisingly consistent in how he defines population thinking. Table 2 reveals the impressive uniformity and convergence that exists across the numerous formulations of population thinking that Mayr offered in his work over a span of half a century.

**Table 2:** Definitions of population thinking offered by Mayr in a variety of contexts over the course of almost 50 years, showing how remarkably consistent he was in his characterization of this notion

Year	Mayr’s Formulation of Population Thinking	Source
1955	“According to this concept no two individuals or biological events are exactly the same and processes in biology can be understood only by a study of variation”	Mayr 1955: 48
1958	“Population thinking is to assume there is no such thing as a type or a norm, only a statistical mean or a mode”	Gerard 1958: 164
1972	“population thinking […] stresses that classes of biological objects constitute populations consisting of uniquely differing individuals”	Mayr 1972: 59
1977	“We call the concept which emphasizes the unique distinctness of every individual *population thinking*”	Mayr 1977: 325
1982	“Population thinkers stress the uniqueness of everything in the organic world. What is important for them is the individual, not the type”	Mayr 1982: 46
1988	“*Population thinking*: Living nature does not consist of types but of variable populations in which each individual is unique”	Mayr 1988: 193
1991	“population thinking, a belief in the reality of variation within a population and in the importance of these individual differences”	Mayr 1991: 74
2001	“An understanding of the […] difference between a class of essentially identical objects and a biopopulation of unique individuals is […] ‘population thinking’”	Mayr 2001: 92
2004	“*Population Thinking*: The realization that in biological populations of sexually reproducing organisms every individual is unique”	Mayr 2004: 223

Mayr, then, did *not* significantly depart from his own canonical characterization of population thinking quoted at the start of this paper (i.e., Mayr 1959a: 2). What he understood by the concept remained constant over time and is quite straightforward. The basic idea is that everything in the living world is fundamentally unique. This, of course, is not to claim that every biological entity is different from every other in *every* respect, but rather that no two biological entities are ever exactly alike in *all* respects. As Mayr himself puts it, each biological individual “has a unique constellation of characteristics […] found in no other individual” (Mayr 1988: 16). It follows from this that biological populations are always heterogenous composites of distinct individualities that can only be treated collectively by means of statistical measures. What population thinking requires is that we do not lose sight of individual differences when dealing with groups of biological entities.

Despite Mayr’s consistency in how he construed population thinking, the term is often understood today in a markedly different way. Many take population thinking to mean that the population—typically understood as an interbreeding community of organisms—is the fundamental unit of biological analysis; it is the level at which one should identify the causes of, and formulate the explanations for, biological phenomena. The population is thus deemed to possess a degree of autonomy over the individuals that constitute it, which means that most of the properties of these individuals are irrelevant to understanding the dynamics of the population as whole. I shall hereafter refer to this viewpoint as *population-level thinking*, in order to avoid confusing it with actual population thinking as understood by Mayr.

I am not the first to notice this. In 2011 population geneticist Jody Hey published an important paper that drew attention to this confusion (Hey 2011). But the problem is more serious than Hey supposed. It is not just population biologists who tend to misunderstand population thinking, but philosophers of biology as well. And the misunderstanding has only become more widespread since the publication of Hey’s paper. Table 3 lists various instances of purported articulations of population thinking in the literature after 2011 that are, in fact, articulations of population-level thinking.^9^

The reason why this confusion is so troubling is that population-thinking and population-level thinking are not only independent ideas but are in some respects at odds with one another. While population thinking emphasizes the particular differences between *individuals* in a population, population-level thinking foregrounds the properties of the *population* as a causally efficacious whole in its own right. The two ideas also have separate histories. Population thinking has long been commonplace among breeders, collectors, and naturalists, whereas population-level thinking is intimately tied to the work of the biometricians and the population geneticists of the early twentieth century. Although both are closely connected with Darwinism, neither was explicitly championed by Darwin himself (who did not even use the term ‘population’ in any of the editions of the *Origin*).

**Table 3:** Recent instances in which the concept of population thinking has been incorrectly characterized as population-level thinking

‘Population Thinking’ Misconstrued as Population-Level Thinking	Source
“In the population thinking characteristic of evolutionary biology, […] [o]ne needs no knowledge of the particular properties of particular individuals. It is only properties of populations that are truly explanatory”	Boulter 2012: 92
“Population thinking cites the structure of a population to explain the properties of a population”	Ereshefsky 2012: 382
“Population thinking recognizes that the unit of evolutionary change is the population […] [It is] applied toward a wide variety of biological phenomena seeking explanations through the lens of population genetics”	Wagner 2016: 3, 4
“‘population thinking’ […] is not a precisely defined term; but in part, it means treating the population as the relevant unit of analysis”	Okasha 2019: 19
“The orthodox interpretation of Modern Synthesis population thinking is that it articulates the causes of evolution that operate exclusively at the level of populations”	Walsh 2019: 228
“population thinking is the basic metaphysics of the ES [Evolutionary Synthesis] […] [It] traces changes in allele frequencies in a population at the micro-evolutionary scale”	Suzuki 2021: 4

Hey (2011) suggests, following Sober (1980), that it is more appropriate to point to Francis Galton—Darwin’s cousin—as the first to articulate the two ideas. In contrast to earlier statisticians like Adolphe Quetelet, Galton interpreted the variability in a population as natural, inevitable, and lawful (instead of as accidental deviations from the population’s typical natural state), and he also criticized the excessive reliance on statistical averages.^10^ At the same time, Galton believed that “[t]he science of heredity is concerned with […] large Populations rather than with individuals, and must treat them as units” (Galton 1889: 35).

As Margaret Morrison (2004) has shown, Karl Pearson—Galton’s protégé—also tried to make room for both ideas. Although he recognized the uniqueness of individuals, he thought that science required statistics; it could not be based on knowledge of individuals alone. Pearson was simultaneously an ontological population thinker and a methodological populational-level thinker. Only with the seminal work of R. A. Fisher (1930) do we have the rise of truly ‘anti-individualist’ population-level thinking of the sort that came to be associated with population genetics. Fisher was greatly inspired by the kinetic theory of gases in his theorizing about the evolutionary dynamics of populations. Just as in physics we can explain the behaviour of a gas without detailed knowledge of the properties of the particles that make it up, Fisher thought that in biology we can explain the evolution of a population without detailed knowledge of the properties of the individuals that constitute it.^11^

What is the source of this confusion? Why is population thinking, as Table 3 shows, so often mischaracterized as population-level thinking? Hey (2011) blames Mayr for his unfortunate choice of words when he coined the term, and there is probably something to this. After all, population thinking is not really about populations, but about *individuals*. For the population thinker, as Mayr (1959a: 2) rather bluntly puts it, “only the individuals of which the populations are composed have reality”. But this is far from obvious. In fact, upon first encountering the term ‘population thinking’, one might reasonably infer a meaning closer to population-level thinking than to any of the related definitions offered by Mayr in Table 2.

Even so, I do not believe that this is the main source of the confusion. I think it is Sober, rather than Mayr, who is most directly responsible for the conflation of population thinking and population-level thinking. As I anticipated at the end of the previous section, a close reading of Sober’s classic paper reveals that while its aim is to provide further support for Mayr’s biological anti-essentialism, Sober does not actually rely on Mayr’s conception of population thinking to do so, as he does not think it is fit for purpose. Just as Sober reframes Mayr’s Platonic essentialism in Aristotelian terms, he also reframes Mayr’s individualist population thinking in populational terms.

Sober’s mistake is to assume that population thinking should reflect the thinking of *population biologists*—such as population geneticists and theoretical ecologists—which is something that Mayr never claims. Indeed, Sober complains, quoting Mayr, that “[i]f “only the individuals of which the population are composed have reality,” it would appear that much of population biology has its head in the clouds. The Lotke-Volterra [sic] equations, for example, describe the interactions of predator and prey *populations*” (Sober 1980: 352).

Although Sober tells his readers at the outset that “our task will be to explicate and explain Mayr’s insight that the shift from essentialist to populationist modes of thinking constituted a shift in the concept of biological reality” (ibid.), what his paper offers is not so much an explication of what Mayr said as a reconstruction of what Sober thinks Mayr *should* have said—one that is partly in conflict with what Mayr *actually* said. This is best exemplified by Sober’s oft-quoted description of the core of population thinking (which is reminiscent of Fisher’s approach to the theoretical study of populations):

The population is an entity, subject to its own forces, and obeying its own laws. The details concerning the individuals who are parts of this whole are pretty much irrelevant. Describing a single individual is as theoretically peripheral to a populationist as describing the motion of a single molecule is to the kinetic theory of gases. In this important sense, population thinking involves *ignoring individuals*: it is holistic, not atomistic. (Sober 1980: 370)

Sober also states that “[p]opulation thinking involves the thesis that population concepts may be legitimized by showing their connections with *each other*, even when they are not reducible to concepts applying at lower levels of organization” (ibid.: 350). In Sober’s analysis, then, population thinking just *is* population-level thinking.^12^ It is not concerned with how the peculiarities of individuals shape the populations they constitute, but with how the structure and dynamics of populations can be accounted for by means of statistically defined population-level properties, quite independently of the properties of individuals. While in Mayr’s population thinking the individual is the explanans when the population is the explanandum, in Sober’s population-level thinking the population is always the explanans *as well as* the explanandum. The population-level thinker invokes population-level features to explain further population-level features. In contrast, the population thinker rejects the notion that the population is an autonomous unit of explanation and that statistical reasoning renders considerations of individuals irrelevant. Far from vindicating the use of statistical explanations in biology (as population-level thinking does), population thinking serves as a powerful reminder of the *limits* of statistical approaches, as these can obscure individual differences within populations. I will return to this point later.

Sober 1980 has long been the gateway to philosophical discussions of population thinking—we should not forget that Sober strategically placed his paper immediately after the abridged version of Mayr 1959a in every edition of his anthology *Conceptual Issues in Evolutionary Biology*^13^—so it is not completely surprising that his reinterpretation of Mayr’s population thinking has been so influential, as Table 3 illustrates. And yet the resulting confusion has been considerable. For example, population thinking is sometimes criticized for neglecting the role of the individual in evolution, which is ironic as that is precisely what it is meant to emphasize. Walsh (2000) has even called for the adoption of ‘individual thinking’ to counteract the adverse effects of Mayr’s population thinking (by which he really means Sober’s population-level thinking), not realizing that he and Mayr are making the same point.^14^ Of course, much of this confusion is a consequence of philosophers not reading Mayr closely enough (or not at all), and relying on Sober’s ‘explication’ of Mayr’s ideas instead.^15^

Still, if what Mayr wanted to emphasize was the uniqueness of *individuals*, why did he call it *population* thinking? I suspect that the answer lies in the closely related notion of variation. Variation is a relational concept—it inherently involves several entities. Although variation describes individual differences, it is a feature of groups, not of individuals. We do not speak of the variation of a tulip, but of the variation across a field of tulips. Hey (2011: 261) is quite right when he remarks that ‘variation thinking’ would have been a better choice of term for the idea that Mayr wanted to convey. At the very least, it is hard to imagine it becoming entangled with population-level thinking to the same regrettable extent. I shall discuss the populational aspect of population thinking in more detail in the next section.

The confusion over the meaning of population thinking has become even more acute in recent years owing to Ariew’s redefinition of the concept. Ariew recognizes the differences between Mayr’s population thinking and Sober’s population-level thinking (though he does not terminologically distinguish the two), but he distances himself from both, as he thinks that they are each grounded in “rather silly metaphysics” regarding what is ‘really real’ (Ariew 2008: 65). He confesses to the reader that “if you are like me, you are still a little confused about which one, individuals or populations, is real” (ibid.: 71), and he proposes instead to reformulate population thinking as a *methodological* strategy for explaining how population-level regularities arise from individual-level differences. Ariew argues that only by reframing population thinking in this way can it capture what was truly groundbreaking about Darwin’s theorizing; neither Mayr nor Sober succeed in doing so with their respective accounts.

There are several problems with Ariew’s account. First, he does not seem very interested in trying to understand the metaphysical issues that exercise Mayr and Sober. What they mean by reality concerns the ability to entertain causal relations. “To be real”, Sober (1980: 371) explains, “is to have causal efficacy; to be unreal is to be a mere artefact of some causal process”. So the issue is about what entities we afford causal efficacy and single out in our explanations. Are population-level properties and events causally efficacious or higher-order effects? I would be surprised if Ariew—of all people—would find such questions “silly”. Second, Ariew is incorrect to set up such a strict division between metaphysical and methodological considerations. Although Mayr and Sober are motivated by the metaphysical issues, their accounts have obvious epistemological implications. Third, it is simply not true that Mayr’s account of population thinking cannot capture Darwin’s theoretical innovations, as I will show in the next section.

Finally, and most fundamentally—and this criticism applies to Sober as much as Ariew—there is something rather peculiar about accusing the person who coins a term of failing to understand that term. It is very different from taking issue with, say, Mayr’s biological species concept, which is just one of countless conceptions of the generic (and ancient) notion of species. The idea of species cannot be said to belong to anyone, but population thinking is very much Mayr’s term. If he coined it because he wanted to emphasize biological uniqueness, why muddy the waters by repurposing it to make it mean something completely different? The more sensible approach, it seems to me, is to preserve the meaning that Mayr chose to give to population thinking and resort to alternative terms, like ‘population-level thinking’, to designate other viewpoints and perspectives—there are actually plenty of such terms readily available in the literature.^16^

## Back to Mayr: The Theoretical Significance of Population Thinking

In the remainder of this paper, I will argue that the uniqueness of biological entities—the ‘essence’ of population thinking, so to speak—has implications that have not been widely recognized, and that it is time that we begin affording due consideration to this critical yet long overlooked feature of the biological domain. But I will begin this section by defending and further substantiating Mayr’s bold claim (noted at the start of the paper) that population thinking captures much of what is most innovative and groundbreaking about Darwin’s theory, and that we can do so *without* abandoning Mayr’s own understanding of the concept (as Sober and Ariew ultimately do).

As I see it, population thinking made two seminal contributions to Darwin’s theorizing. The first is that it provided a new way of conceptualizing the dynamics of populational change, and the second is that it prompted a reconsideration of how to explain patterns of similarity and difference between individuals. Let us take each of these in turn.

**Fig. 2:**
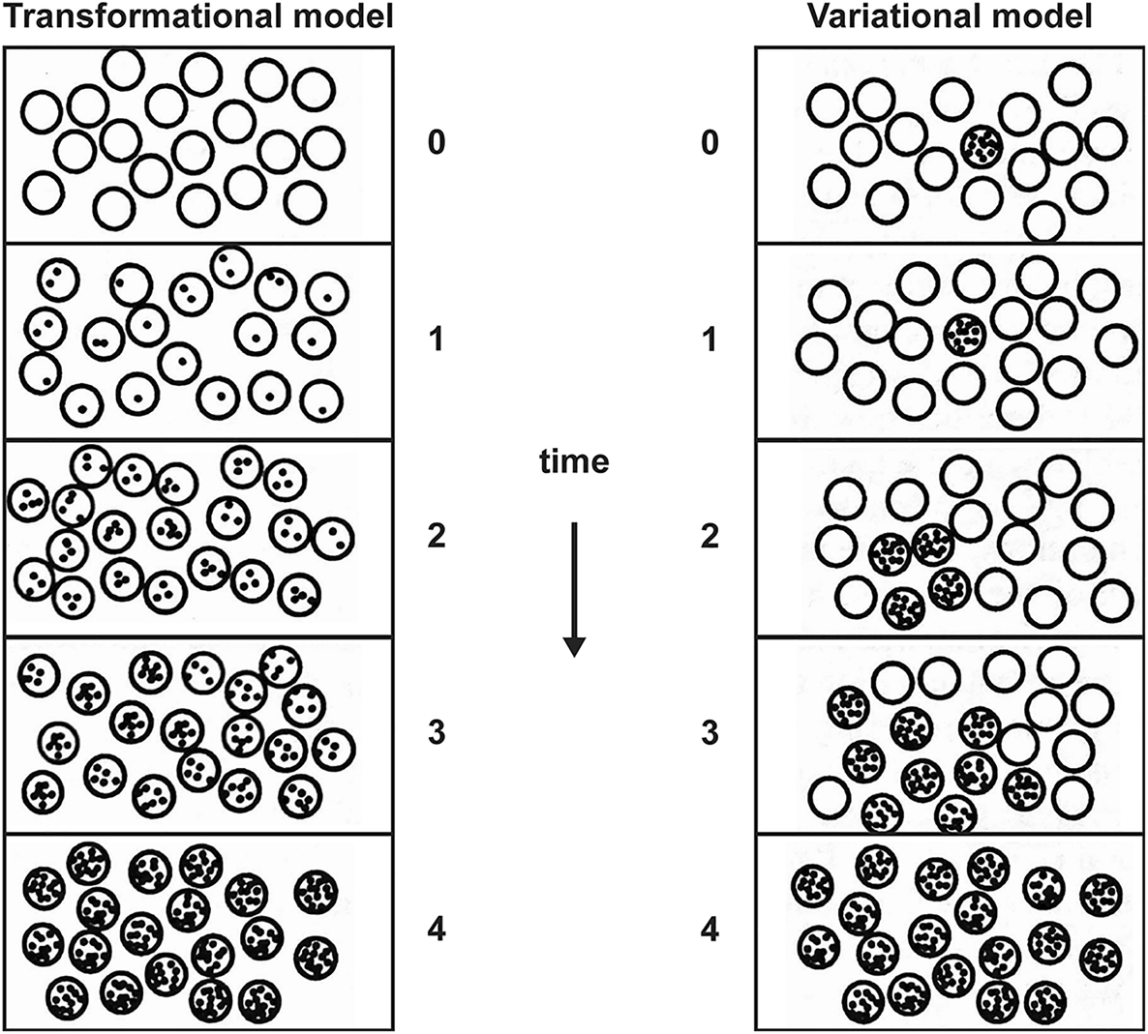
Transformational versus variational models of populational change. Individuals undergoing change are represented by shaded circles, with the intensity of the shading indicating the extent of change. The numbered phases in the evolution of each population can reflect either successive generations or temporal stages within a single generation (after Jablonka and Lamb 1995)

The first contribution is best explained by invoking the distinction between *transformational* and *variational* models of change in a population, illustrated in Fig. 2.^17^ Before Darwin, all theories of change were transformational. In transformational theories, a population changes over time because every member of the population individually undergoes the same gradual transformation during its life history. This transformation can be prompted by environmental influences, or it can be intrinsically goal-directed. Lamarckian evolution is a paradigmatic example of a transformational theory, as it postulates that each organism in a species individually undergoes the same adaptive changes, which are then transmitted to members of the next generation, which in turn continue to increase their adaptive fit to the environment. Our modern biological theories of development and of aging are also transformational, though all the changes in the population occur within a single generation. In astrophysics, our conception of stellar evolution is transformational as well: the universe evolves over time because every star within it is undergoing the same ordered sequence of well-defined stages.

In contrast, Darwin’s theory of evolution is based on a variational model of change. The population changes not because each individual undergoes a parallel transformation over its lifetime, but because variation is produced anew in every generation and some variants leave more offspring than others. The population changes as a whole because the turnover of all of its members from one generation to the next results in modifications in the proportional representation of the different variants that make it up, even though the variants themselves do not fundamentally change in their individual properties.

What made Darwin’s theory of evolution so groundbreaking is that it shifted the locus of change from the individual to the population, and it stripped the evolutionary process from its teleological character. In transformational theories like Lamarck’s, it is the *individuals* that evolve, and they do so in a definite direction. Populational change is dependent on every individual successfully passing through a series of transformative stages, each of which is a prerequisite for reaching the next stage in the process. There are no shortcuts, no reordering of the transformation, and only one possible end to the process. In Darwin’s variational theory, however, it is not the individuals that evolve but rather the *population* as a whole. Out of static variation among individuals in space, a dynamic process of populational change emerges in time. The resulting evolution is not necessarily progressive, as it does not strive towards perfection or any other predefined goal. It is opportunistic, and therefore unpredictable.^18^

It should be clear how population thinking underlies this picture. The epistemological transition from a transformational to a variational conception of populational change marks, as Lewontin (2001: 64) observes, “a change in the object of study from the average or modal properties of groups to the variation between individuals within them. That is, *variation itself* is the proper object of biological study”. While in transformational theories one can infer the ‘type’ or the ‘norm’ of a population from a consideration of its individual members, in variational theories any statistically derived properties of the population represent abstractions from the existing variability between its members. This pervasive and irreducible variability—which reflects the uniqueness of biological individuals—provides the raw material for Darwinian evolution. This is what Mayr (1988: 264) means when he states that it was “population thinking that made the theory of natural selection possible”. In Darwin’s theory, natural selection converts individual-level variations into population-level changes. A perfectly homogeneous population could not possibly evolve by natural selection.

The second major contribution that population thinking made to Darwin’s theorizing is that it challenged the assumption that individual *similarities* are more fundamental than individual *differences*.^19^ By making variation the cornerstone of his theory, Darwin came to appreciate that the respects in which individuals differ from each other are at least as—if not more—significant than the respects in which they resemble one another. Much of the commotion caused by Mayr’s incendiary invocation of Plato in his account of the biological essentialism that Darwinism overthrew probably could have been avoided if it had been interpreted along these more sober lines. To the extent that anything like an essentialist or typological tradition existed in pre-Darwinian biology (and surely something did), a relatively uncontroversial way to characterize it would be to note that it regarded the features shared by members of a biological kind to be ontologically more significant than the features they do not share. The fact that members of a species are never identical was of course recognized, but this could be explained in Platonic terms by maintaining that no individual perfectly embodies the ideal of the kind that it reflects (as indicated by Mayr)—or in Aristotelian terms by supposing that the differences between members of a kind are the consequence of interferences or perturbations in the natural state that is characteristic of that kind (as indicated by Sober). From this perspective, variation among conspecifics is just meaningless noise that gets in the way of our ability to discover the underlying nature of the kind to which they belong. To explain what it is for individuals to be of the kind that they are is to learn to *see through* the confusing variation that superficially sets them apart and thereby uncover their deeper sameness.

Construed in this way, it becomes easier to understand this crucial and novel aspect of the Darwinian worldview—and why it was population thinking that made it possible. To acknowledge that every biological individual is unique is to reject the ontological primacy of sameness over difference. What makes organisms alike does not take causal precedence over what makes them different.^20^ Differences between individuals are as ‘normal’ and as ‘natural’ as their similarities. The former are not accidental byproducts of the latter. If anything, the reverse is closer to the truth. Because natural selection is a sorting process that raises the proportional representation of some variants over others in a population, it will tend to make the population more uniform with respect to certain traits (at least for a time). In other words, it is similarity that arises from difference, not the other way round. Variation is a primary, irreducible, and inescapable feature of the living world.

These two key contributions already justify, I think, the central role that Mayr affords to population thinking in the Darwinian revolution, and yet its importance for evolutionary biology does not end there. Take the aforementioned species-as-individuals thesis, popularized by Ghiselin (1974) and Hull (1976). This widely discussed philosophical thesis is grounded in population thinking. Paradoxically, however, it also reflects a *failure* to fully come to terms with population thinking. Let me explain.

Ghiselin’s motivation for advancing the species-as-individuals thesis was to expose the inadequacies of, and offer an alternative to, the longstanding essentialist conception of species as *classes*. Species taxa are not classes because species membership is not determined by the possession of essential properties. To regard species taxa as individuals is to recognize that they are: (i) concrete particulars (rather than abstract universals) designated by proper names, (ii) spatiotemporally restricted, (iii) internally cohesive, and (iv) capable of splitting (through speciation), fusing (by hybridization), evolving, and becoming extinct. Classes, as conventionally understood, lack all of these properties. Still, individuals are not the only kinds of entities that display them; biological populations do as well.

Now Ghiselin was evidently understanding the term ‘individual’ in a *very* general sense—consistent with how logicians and metaphysicians have traditionally used it, which is as a contrast to the idea of a class. But this technical philosophical meaning can be confusing as it is not what most people, including most biologists, have in mind when they think of an individual. For them, individuals are singular particulars (like organisms), not groups of particulars (like species), so referring to species as individuals is a less-than-ideal alternative to the flawed essentialist conception of them as classes (also because species do not exhibit the same degree of internal cohesion as paradigmatic individuals like organisms). ‘Population’ would have been the more suitable terminological choice here, as it captures everything that ‘individual’ does in relation to the crucial contrast with ‘class’ without conflicting with how the term is ordinarily used by biologists (and in everyday life).

Mayr (1987) suggests that the reason the non-class conception of species has come to be known as the species-as-*individuals* thesis—and not the species-as-*populations* thesis—is that philosophers (especially those trained in the physical or formal sciences) tend to think of populations logically as ‘sets’ (e.g., Kitcher 1984) in a way that drastically underestimates the inherent variability that is so characteristic of *biological* populations, so the term ‘individual’ might have seemed the less ambiguous choice for that reason. If this is right, then a more widespread recognition *among philosophers* of the importance of population thinking in biology might have made Ghiselin’s awkward terminological appeal to ‘individuals’ unnecessary in the protracted debate over the ontological status of species taxa, as there would have been less of a risk of philosophers inadvertently confusing biological populations with the more homogeneous kinds of populations, more akin to sets, typically found in other domains.^21^

But leaving this issue aside, there are good reasons to suppose that population thinking has not even permeated all areas of *biology*. This is why Mayr’s critiques of typology and essentialism are not confined to the historical record. Some of the assumptions of those views—as well as the methodological habits that derive from them—are alive and well in biology today, despite being antithetical to population thinking. Consider, for instance, the familiar habit that Mayr (1976: 421) discusses to speak of ‘*the* mouse’ or ‘*the* fruit fly’ (or, to use a more contemporary example, ‘*the* human genome’) as if these referred to real entities that can be directly studied, instead of as convenient abstractions, which is what they are. Recent commentators, it seems to me, have been a bit unfair in portraying Mayr as tilting at windmills here. For one thing, he is far from the only prominent evolutionary biologist to have made these criticisms—or even to controversially root them in Plato’s metaphysics. For example, Lewontin, who is less prone to hyperbole than Mayr, has nevertheless remarked along very similar lines that

Biology remains in many ways obdurately Platonic. Developmental biologists are so fascinated with how an egg turns into a chicken that they have ignored the critical fact that every egg turns into a different chicken and that each chicken’s right side is different in an unpredictable way from its left. Neurobiologists want to know how the brain works, but they don’t say whose brain. (Lewontin 2001: 67)

Gould also shares Mayr’s concerns. Like Mayr, he has argued at length that “we are still suffering from a legacy as old as Plato, a tendency to abstract a single ideal or average as the ‘essence’ of a system, and to devalue or ignore variation among the individuals that constitute the full population” (Gould 1996: 40). Despite living “[i]n Darwin’s post-Platonic world”, Gould adds, “we continue to […] regard variation as a pool of inconsequential happenstances, valuable largely because we can use the spread to calculate an average, which we may then regard as a best approach to an essence” (ibid.: 41).

Now we may quibble about the somewhat gratuitous references to Plato in these remarks, but to fixate on that—as commentators often have—is to completely miss the point that Mayr (and Lewontin and Gould) was trying to make, which is that there is a *real* problem in contemporary biology resulting from the common tendency to disregard differences among members of a population. For evolutionary biologists and geneticists, intraspecific variation is, of course, necessarily at the center of attention, but this is far from the case in many other areas of biology, where it tends to get downplayed—if not dismissed altogether—in favor of a focus on average values.

Physiologist Albert Bennett (1987: 150) memorably dubbed this unfortunate predicament “the tyranny of the Golden Mean”, which “restricts our vision of the data and narrows our conceptual framework so that we cannot take advantage of all the analytical possibilities of biological variability”. Here is his more detailed description of the problem:

The framework of physiological studies implicitly emphasizes the description and analysis of central tendency. Depending on the data, this involves calculation of mean values or the development of […] regression equations. After these values are determined, they take on a life of their own and become the only point of analysis and comparison. The complete breadth of biological variation determined in the investigation then is forgotten. Measures of variability (e.g., variance, standard deviation) are calculated and reported only to stipulate confidence limits about the mean or slope of the regression line […]. The variability inherent in the original data is seen only as ‘noise’, through which the ‘true’ value of the central tendency can be glimpsed with appropriate statistical techniques. (Bennett 1987: 148)

Bennett argued that physiologists’ dismissal of variation is licensed by the conviction that it is either: (a) not real (i.e., it is due to measurement and experimental error) or (b) ‘atypical’ or ‘abnormal’ and therefore not significant (suggesting that the essentialist natural state model is still very much engrained in physiology). To support his claims, Bennett examined all papers published during 1985 in three leading physiology journals and found that not even 5% reported—let alone discussed—the range of values of the obtained data. These results were replicated twenty years later in the specific physiological field of endocrinology, “confirming that most authors continue to use variance in their data simply to provide confidence limits about the estimated mean values (Bennett 1987), rather than exploring this variance itself. Thus, there is a paucity of studies on inter-individual variation with which to work” (Williams 2008: 1688).

Nevertheless, it is becoming apparent to most physiologists that intraspecific variation is both more real and more meaningful than was previously supposed. Measurement and experimental error accounts for only a fraction of the extraordinary degree of variability that is consistently observed in most physiological studies, regardless of context and of temporal scale, and which manifests itself as developmental, morphological, behavioural, and neurological differences that often have far-reaching ecological and evolutionary consequences (see, e.g., Barash 1997; Hayes and Jenkins 1997; Dall et al. 2012; Wolf and Weissing 2012; Roche et al. 2016).

**Fig. 3:**
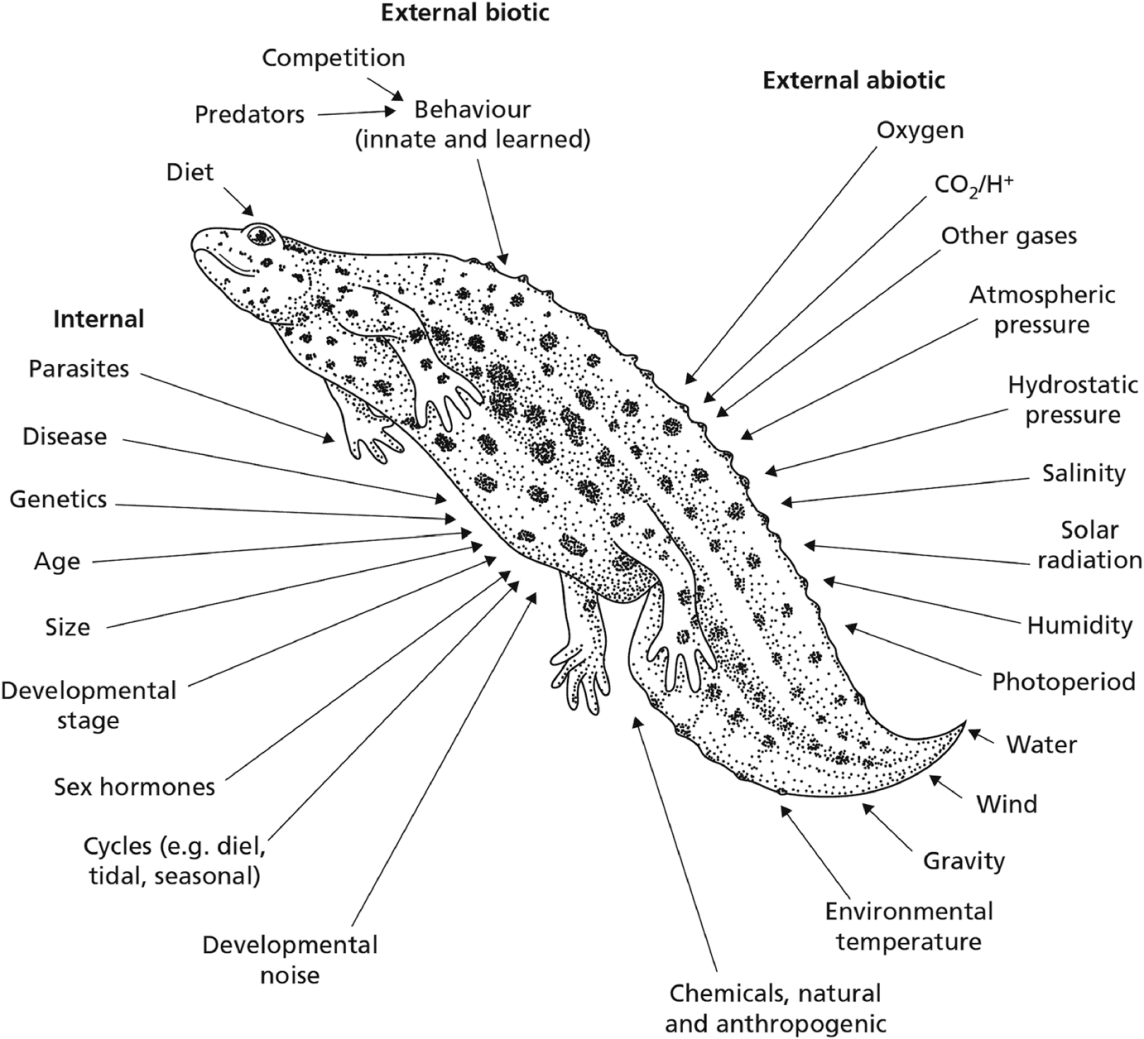
Range of internal and external biotic and abiotic factors that can result in individual variation in a particular physiological trait. The quality, quantity, duration of exposure, and/or rate of change in each of these factors can affect the extent and nature of the variation (after Spicer and Gaston 1999)

Figure 3, taken from the textbook *Physiological Diversity and its Ecological Implications* (Spicer and Gaston 1999), showcases the impressively wide array of factors that can lead to differences among conspecifics. Of particular note is the fact that genetics is listed as just one of the many sources of this phenotypic diversity, despite having received the lion’s share of the attention (at least among evolutionary biologists and geneticists, and arguably among developmental biologists as well) over the past hundred years. The overall message emerging from this research could not be any clearer: *every organism is physiologically unique*,* and we ignore this variation at our peril*.

I come now to an aspect of Mayr’s conception of population thinking that has been strangely overlooked by almost all commentators, which is that it was never meant to apply to just organisms. Organisms (and other biological individuals) are the most conspicuous biological entities that are unique, but they are definitely not the only ones. We have already seen that the species-as-individuals thesis implies that uniqueness is likewise a feature of demes, populations, species, and all higher taxa. What is perhaps less obvious is that Mayr always considered uniqueness to also be a feature of biological entities at the sub-organismic and even the sub-cellular level.^22^

In his canonical articulation of population thinking of 1959, quoted at the start of this paper, Mayr states that “[a]ll organisms *and organic phenomena* are composed of unique features”. In case the reader misses it, he repeats the point in the following sentence by referring to biological “[i]ndividuals, *or any kind of organic entities*” (Mayr 1959a: 2, my emphasis). In other words, there is uniqueness in the biological hierarchy not just all the way up but also *all the way down*. Mayr makes this point more explicitly in *The Growth of Biological Thought*:

This uniqueness is true not only for individuals but even for stages in the life cycle of any individual, and for aggregations of individuals whether they be demes, species, or plant and animal associations. Considering the large number of genes that are either turned on or turned off in a given cell, it is quite possible that *not even any two cells in the body are completely identical*. This uniqueness of biological individuals means that we must approach groups of biological entities in a very different spirit from the way we deal with groups of identical inorganic entities. This is the basic meaning of population thinking. (Mayr 1982: 46–47, my emphasis)

In subsequent writings, Mayr reiterates the idea that “[a]mong the millions of cells of an organisms [sic] no two are probably exactly identical, owing to the diverse activity (suppression and activation) of regulatory genes” (Mayr 1985: 55), indicating also that “[i]n the living world, uniqueness is seen *even at the molecular level*,* in the form of DNA or RNA*” (Mayr 1988: 16, my emphasis).

To the best of my knowledge, the bearing of population thinking on microscopic entities has never been carefully examined, by Mayr or anyone else. Given Mayr’s own naturalist predilections, as well as the disciplinary contexts in which population thinking has hitherto been considered, one could almost be forgiven for supposing that population thinking is simply unnecessary at this level.^38^ It turns out that the opposite is the case. As we will see in the next section, it is at the level of cells and macromolecules that the implications of uniqueness are most striking and arguably also most consequential.

## The Rise of Population Thinking in Molecular Biology

The methodological challenges involved in studying the molecular basis of cellular behaviour are so formidable that they have forced molecular biologists to approach their subject as if they were population biologists. Consider the all-important process of *gene expression*. Until recently, this phenomenon had to be studied by interrogating lots of cells all at once. The reason for this is that when using conventional molecular biology techniques (e.g., Northern blots, microarrays, reverse transcriptase-polymerase chain reaction), the only way to amass enough gene product to reach a detectable threshold is to grind up vast numbers of isogenic (i.e., genetically identical) cells grown under the same conditions and then measure the amounts of the relevant mRNA or protein in the homogenate. Thus, although the goal is to understand the behaviour of an *individual* cell, one proceeds by studying the behaviour of a *population* of cells. The consequence of this is that the specific behavioural patterns of individual cells are averaged out across the entire population, and—as we have already seen—this can conceal differences between its members.

For a long time, this methodological limitation was not considered a problem because, as Sui Huang puts it, “molecular biologists habitually assume uniformity of the cell populations that serve as starting material for experimental analysis” (Huang 2009: 3853). If all cells are presumed to be identical and one is therefore dealing with a homogeneous population, then one can confidently infer that the average behaviour of the population as a whole accurately reflects the individual behaviour of each cell in that population. This essentialist assumption is grounded in the conviction that the cell is fundamentally determined by its genetic makeup, which acts as a sort of blueprint or program for its structure and operation. Accordingly, cells with the same genes exposed to the same environmental conditions are expected to behave almost identically—just as cell phones, laptops, or cars manufactured in the same assembly line according to the same model design will behave almost identically.^23^

These longstanding beliefs are now increasingly being called into question. Major technological advances and experimental innovations have granted us unprecedented access to the real-time dynamics of individual molecules inside single cells (Zlatanova and van Holde 2006; Deniz et al. 2008; Kapanadis and Strick 2009; Leake 2013). And as molecular biologists have begun to monitor biological processes on a cell-by-cell and molecule-by-molecule basis, they have come to the surprising realization that there exists “a hidden world beneath population averages” (Altschuler and Wu 2010: 559). Being able to measure the entire distribution of cellular behaviours across a population, as opposed to merely relying on the average behaviour of the population as a whole, has unexpectedly revealed that even isogenic cells behave quite differently from one another. Just as there is no such thing as a perfectly homogeneous population of organisms, there is no such thing as a perfectly homogeneous population of cells.

The shift in our understanding of gene expression has been one of the catalysts of this realization. It has long been known that when cells are treated with varying intensities of an inducer and the gene product (mRNA or protein) corresponding to a specific gene is assayed, the level of gene product changes in a smooth, dose-dependent manner. Specifically, a gradual increase in the concentration of the inducer results in a proportional increase in the expression of the gene. To make sense of this, it was postulated that cells adjust their rate of expression of a responsive gene progressively and linearly from zero to its maximum output in direct proportion to a rising concentration of an inducer. This came to be known as the *graded model* of gene expression, and it remained the dominant view until the end of the last century.

Recently, however, single-cell studies have shown that raising the concentration of an inducer in an isogenic population typically does not lead to a corresponding increase in the rate of transcription in every cell in the population (as hypothesized by the graded model), but rather results in the recruitment of a rising number of cells that respond spontaneously in an all-or-nothing fashion once their unique activation thresholds have been reached. In other words, in each cell of the population the target gene is either maximally expressed, or it is not expressed at all, and the probability of its expression in each cell rises as the concentration of the inducer increases. In addition, once a cell begins to express the gene, its rate of expression remains unaffected by further increases in the concentration of the inducer. With regard to each of its genes, every cell appears to exist in one of two functional states: it is either ‘on’ or ‘off’. This is known as the *stochastic model* of gene expression, and it has become very widely accepted in recent years. (For a more detailed discussion, see Nicholson 2019.)

**Fig. 4:**
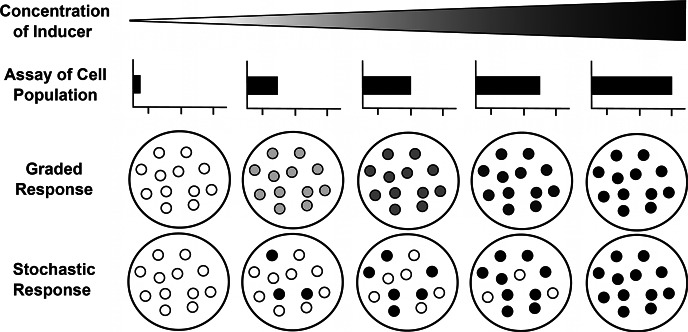
Graded versus stochastic models of gene expression. If gene expression is measured by assaying the whole population, it is not possible to discriminate stochastic from graded transcriptional responses, as both are consistent with population-level observations. Single-molecule methods, however, have recently enabled gene expression to be studied on a cell-by-cell basis, and this has revealed that most cells exhibit an all-or-nothing stochastic expression pattern (after Nicholson 2019)

This conflict between graded and stochastic models of gene expression presents us with a classic case of underdetermination of theory by data. When the only way to measure gene expression in a cell was to assay the total amount of mRNA or protein produced by an entire population of cells, it was simply not possible to discriminate between the two competing models. Indeed, the familiar observation that progressively raising the concentration of an inducer results in a proportional increase in the expression of the corresponding gene is equally compatible with both models, as Fig. 4 shows. The level of gene expression in the population as a whole could reflect similar levels of gene expression in all cells (as postulated by the graded model) or the statistical mean of different subsets of cells either expressing or not expressing the gene (as postulated by the stochastic model). It is only with the advent of single-molecule methods that it has become possible to examine gene expression on a cell-by-cell basis, and thereby distinguish the two models experimentally.^24^

The example of gene expression illustrates rather dramatically what can happen when one is forced to average out data across a population due to methodological limitations. The longstanding dependence on population-averaging methods has inadvertently driven molecular biologists to rely on what Jeffrey Levsky and Robert Singer (2003: 4) fittingly call the ‘average cell’, which they describe as “a statistical contrivance for representing biological knowledge beyond the limits of detection”. But, as these authors go on to point out, the adoption of single-molecule methods has shown that the average cell that is routinely extrapolated from experimental studies *does not exist*. Variability is everywhere in the cellular world.

Seeing molecular biologists denounce ‘the myth of the average cell’ (Levsky and Singer 2003), one cannot help but be reminded of the myrmecologists who are proclaiming ‘the demise of the standard ant’ (Heinze 2008), the aforementioned physiologists who are protesting ‘the tyranny of the Golden Mean’ (Williams 2008), or the ecologists who “advocate moving beyond the mean field theory in community ecology by examining the structure of intraspecific variability in actual communities” (Violle et al. 2012: 250). This extraordinary concurrence of convergent declarations in disparate biological disciplines shows that population thinking is seeping into areas of biology that have hitherto remained largely untouched by it. And yet, there is something especially consequential about the prominence that population thinking is acquiring in molecular biology. Let me elaborate on this point.

For evolutionary biologists and geneticists (the kinds of biologists most accustomed to population thinking), *genetic differences* are by far the most important source of variation in a population. The influence of the environment is certainly recognized, but it is seldom afforded the same theoretical consideration. The situation is a bit different for physiologists, who typically do take into account numerous aspects of the organism’s internal and external milieu in addition to genetics when explaining phenotypic differences among conspecifics, as Fig. 3 illustrates. But what is particularly remarkable about the rise of population thinking in molecular biology is that it has been prompted by the recognition that even when we are dealing with a population of *genetically identical* individuals subject to *identical environmental conditions*, we *still* find considerable variation in their form, function, and behaviour. This variation is generally referred to as ‘non-genetic heterogeneity’, and in recent years it has become an important object of study in its own right (Huang 2009; Brock et al. 2009; Altschuler and Wu 2010).

Initially, the discovery of non-genetic heterogeneity was dismissed as *noise*, which is precisely how an essentialist thinks about the nature of variation; that is, as an unwanted random disturbance that thwarts a system or a process from realizing its natural state (recall Sober 1980). The adoption of population thinking has led to the realization that non-genetic heterogeneity is not only not ‘abnormal’ or ‘unnatural’, but that, on the contrary, it is precisely what we should expect to find given the physical scale at which biological processes inside the cell take place.

The reason why no two cells ever behave or respond to a stimulus in exactly the same way—even when they are genetically identical and are grown in the same environment—is that the molecular processes that underlie cellular behaviours are inherently *probabilistic*, as they are inevitably exposed to the stochastic dynamics of Brownian motion (which, of course, is true for *any* molecular interaction subject to thermal agitation). These processes are extremely intricate and depend on many different kinds of randomly moving molecules fortuitously coming together in exactly the right way, at exactly the right time, and in exactly the right order. And because most of these kinds of molecules are known to be present in the cell in very low copy numbers (Xie et al. 2008), it is not possible to appeal to the law of large numbers—as we do in classical test-tube biochemistry—to reliably predict the exact outcome or timing of these processes. The stochasticity of molecular interactions generates cell-to-cell variability in yet other ways; for example, by resulting in the uneven partitioning of cytoplasmic contents during cell division (Huh and Paulsson 2011). These are just some of the factors responsible for the heterogeneity that is consistently observed in isogenic cell populations.

A crucial milestone in the study of non-genetic heterogeneity has been the realization that it performs a variety of critical functions (Eldar and Elowitz 2010; Balázsi et al. 2011; Dueck et al. 2015). In microbial biofilms it is a key generator of phenotypic diversity, which enables them to adapt rapidly to changing environmental conditions. And in eukaryotic cells, among other things, it helps determine cell fate decisions during development, thereby shaping the way that embryonic regions differentiate. Non-genetic heterogeneity has even been linked to the resilience of tumors in counteracting the effects of chemotherapy, which limits the efficacy of target-selective drugs (Brock et al. 2009). In general, we know that more heterogeneous cell populations are more robust, and they adapt, grow, and evolve faster than less heterogeneous populations. Far from being a nuisance that cells must tolerate (as the term ‘noise’ misleadingly suggests), non-genetic heterogeneity is an asset that cells exploit in myriad ways.

Another striking discovery prompted by the use of single-molecule methods is that non-genetic heterogeneity is not only a feature of cells but also of organelles and other subcellular units (Chang and Marshall 2017). Uniqueness, as I anticipated earlier, is a feature of biological entities *at every level* as we move down the biological hierarchy. At the organelle level, there is heterogeneity arising from stochastic fluctuations in the cell cycle, as well as in the rates of organelle biogenesis, division, and degradation. Macromolecular assemblies such as ribosomes likewise exhibit a high degree of heterogeneity. Although “[w]e are told that all electrons are identical”, Ignacio Tinoco and Ruben Gonzalez write, “it is less clear that all *Escherichia coli* ribosomes are the same” (Tinoco and Gonzalez 2011: 1205). In a population of ribosomes, there will be differences in sequence, composition, covalent modification, bound ligands, and so forth. Yet even in a population of structurally identical ribosomes, thermal agitation will still deeply impact the series of conformational changes they each undergo, invariably leading to substantial differences in their operation. As Peter Moore (2012) has remarked, it is most unlikely that even a *single* ribosome ever performs the same ‘random walk’ twice as it elongates a polypeptide.^25^

Similar considerations apply to protein complexes. We have learned, for instance, that there is immense variability in the size and composition of protein complexes involved in intracellular signaling, which can in turn undergo numerous reversible post-translational modifications (e.g., phosphorylations) in ways that drastically alter their individual conformations and activities (Mayer et al. 2009). It is becoming increasingly clear that receptor complexes, adhesion complexes, mRNA splicing complexes, trafficking intermediates, and many other kinds of protein associations do not exist in the cell as structurally homogeneous, stable assemblies of fixed subunits mechanically performing the same predefined sequence of structural rearrangements, but as extremely diverse, transient ensembles of ever-changing subunits stochastically flickering between alternative conformations. It has even been suggested that we should conceptualize each kind of complex not as a single entity but as a “probability cloud of an almost infinite number of possible states, each of which may differ in its biological activity” (ibid.: 81.2).

Molecular biologists have ultimately come to appreciate that there is heterogeneity all the way down to the level of individual macromolecules. What we traditionally referred to—in the singular—as *the* conformation of a particular type of protein is actually best described—in the plural—as an array of well-defined configurations separated by low-energy barriers that each token protein samples by means of stochastic fluctuations. Any population of ostensibly identical proteins is really a heterogeneous mixture of macromolecules displaying slightly different conformations. Moreover, many proteins, known as ‘intrinsically disordered proteins’, do not have an ordered conformation *at all*, but instead roam the cell as unfolded polypeptide chains capable of binding to a wide range of substrates (Wright and Dyson 2015).^26^ Promiscuity rather than specificity is the rule for most proteins (including enzymes): what a protein does is as much a product of its cellular milieu as its amino acid sequence (Nobeli et al. 2009). In fact, the same polypeptide chain can partake in a variety of functions depending on when and where it is expressed, and on what partners it binds to—a phenomenon that has been dubbed ‘moonlighting’ (Jeffery 2003). (Again, see Nicholson 2019 for a more detailed discussion.)

Overall, the widespread adoption of single-molecule methods in molecular biology has resulted in a spectacular vindication of population thinking. Note that it was not always obvious that it would turn out to be so. Single-molecule studies *could* have simply corroborated the findings arrived at by conventional population-averaging methods. Part of what makes the sudden rise of population thinking in molecular biology so remarkable is that it has been, to a large extent, unexpected.^27^

**Fig. 5:**
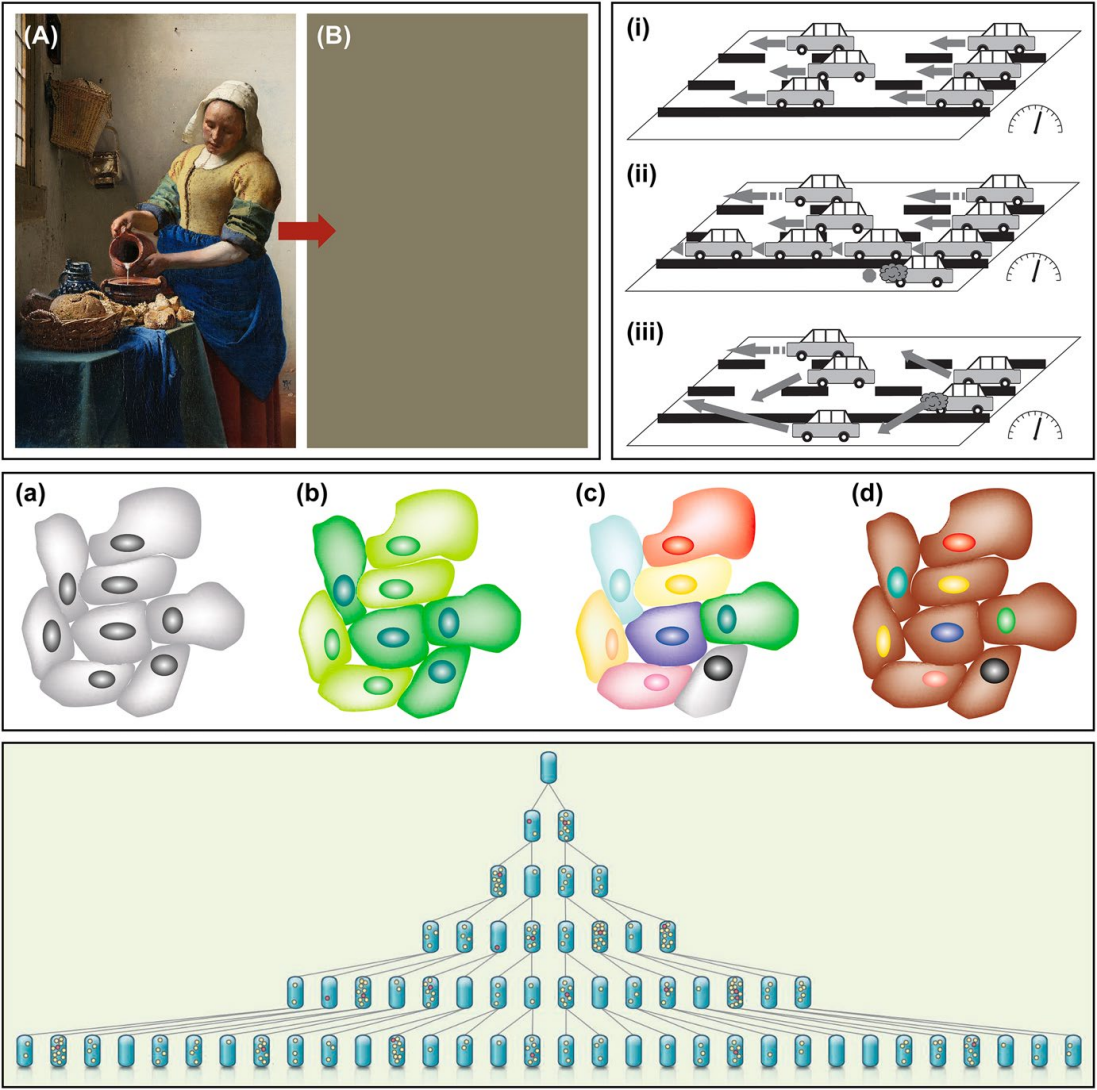
Conveying biological uniqueness at the microscopic scale. In the top left panel, **(B)** is the image we obtain when we average out the properties of all the pixels in image **(A)** (after Mayer et al. 2009). The top right panel shows how measuring the average speed of cars in traffic does not allow us to determine whether the cars are: **(i)** moving at the same speed, **(ii)** moving at different speeds, or **(iii)** changing their speed and direction (after Knight 2008). The middle panel depicts the steps in the evolution of our understanding of cell populations: **(a)** all cells behave the same, **(b)** each cell is either ‘on’ or ‘off’ with respect to a given gene, **(c)** no two cells express all their genes in the same way, and **(d)** post-transcriptional controls help offset cell-to-cell variability (after Levsky and Singer 2003). The bottom panel illustrates how the unequal distribution of organelles and macromolecules during cycles of cell division generates non-genetic heterogeneity at the population level (after Tyagi 2010)

It is quite fascinating to observe how molecular biologists are coming to terms with this ongoing revolution in their field. Figure 5 brings together a selection of rather striking illustrations from various scientific publications that give a sense of how molecular biologists have been trying to visualize and graphically represent the causes and consequences of this revolution. The top left and right panels offer two very different analogies that show how the reliance on results obtained by population-averaged methods has led to the critical loss of information about individual cells and macromolecules. The middle panel depicts the evolution in our conceptualization of cellular heterogeneity, and the bottom panel illustrates the asymmetrical partitioning of cytoplasmic contents during cell division, which, as I indicated above, is one the main sources of non-genetic heterogeneity in cell populations.

I have suggested in this section that the recognition of uniqueness is more consequential at the level of cells and macromolecules than at the more familiar level of organisms. One reason is that at this level it becomes abundantly clear that genetic variation is not the sole, or even the main, source of the uniqueness that makes population thinking so necessary, which is what evolutionary biologists, including Mayr, have traditionally supposed.^28^ The overwhelming amount of variation that has been discovered at the microscopic scale makes population thinking an *even more* central concept in biology than Mayr could have possibly imagined from his lifelong study of birds and his successive careers as a naturalist, museum curator, organismal biologist, and evolutionary theorist.

Moreover, because the populations handled by molecular biologists are a dozen or more orders of magnitude larger than the ones typically studied by organismal population biologists, the amount of lost information pertaining to individual differences is vastly compounded as well. The implications are potentially enormous. It might well be that, as we continue to devote more attention to characterizing not just individual cells but also individual molecules inside individual cells, we will soon find ourselves in the situation of having to fundamentally reconsider our understanding of even the most basic cellular processes.

The population thinking revolution in molecular biology is clearly in full swing, but it is far from complete. One enduring challenge is the continued reticence on the part of some molecular biologists to accept randomness (i.e., stochasticity) as a genuine feature of the biological domain, and as a critical explanatory resource. Thomas Heams (2014) has perceptively observed that what we have at present is a sort of ‘schizophrenic biology’, in the sense that while evolutionary biologists are perfectly comfortable acknowledging the importance of random events (e.g., mutations) and random processes (e.g., genetic drift), many molecular biologists continue to be uneasy about attributing biological functions to randomness.^29^

More generally, there has been virtually no recognition that molecular biologists stand to benefit from adopting a more explicitly Darwinian outlook when interpreting the findings of single-molecule studies and reflecting on the role of individuals in biological populations. All too often, when molecular biologists make provocative claims like “biological variability is less difficult to explain than commonality” (Levsky and Singer 2003: 5), they are unfortunately not aware that proposals of this kind have long been the subject of discussion in evolutionary theory, as we saw in the previous section. One notable exception to this general lack of consideration of Darwinian ideas can be found in the concluding remarks of an opinion piece in *Nature Reviews Genetics* dating back to 2001, which I quote below in full:

Isaac Newton might have liked the neat view of biological systems made up of dedicated components, with causal roles that can be studied in isolation, and in which particular starting conditions give rise to uniquely predictable responses. Charles Darwin, by contrast, might have felt more at home with the idea of a complex, emergent system made up of *many non-identical components*, with non-exclusive roles, non-exclusive relationships, several ways of producing any given output, and a great deal of slop along the way. *We have been Newtonians for the past several decades in our thinking about gene action. It is time to become Darwinians*. (Greenspan 2001: 386, my emphasis)

## Population Thinking, Uniqueness, and the Autonomy of Biology

Let us now take a closer look, in this final section, at some aspects of population thinking that we have not yet had the opportunity to consider pertaining to its historical origins, its philosophical foundations, and its bearing on the question of what makes biology different from physics.

First of all, where does population thinking actually come from? Back in Sect. 4, we saw that the idea is usually associated—and with good reason—with Darwin’s theory. However, although Darwin did emphasize in the *Origin* that “individual differences are highly important for us, as they afford materials for natural selection to accumulate” (Darwin 1859: 45), even Mayr admits that Darwin was not the first to recognize or assume the uniqueness of biological individuals, and that he probably came to appreciate its significance from his careful study of the animal breeding literature (Mayr 1977).^30^ The endless availability of variation has always been taken for granted by breeders and horticulturists, who are adept at picking out minute differences between individuals in a population. The observation that shepherds can tell their sheep apart is so ancient as to be proverbial. Ultimately, the presence of uniqueness is one of the most commonplace, everyday reminders of human experience. “Who is not aware”, Mayr (1982: 487) asks rhetorically in *The Growth of Biological Thought*, “that no two human beings are identical, nor any two dogs or horses?”. This framing would suggest that the roots of population thinking stretch back to the first attempts by the earliest humans to make sense of the natural world.

Yet only a few pages later, Mayr oddly declares, without evidence, that “[p]opulation thinking was virtually nonexistent prior to 1800” (ibid.: 490; see also Mayr 1976: 26). I suspect that the reason for this is that he wants to portray population thinking as an empirically grounded rejection of the metaphysically motivated promotion of typological essentialism by pre-Darwinian biologists. This is why he insists on depicting Darwin and Alfred Russel Wallace—rather implausibly—as “two English amateurs” that managed to avoid becoming influenced by “philosophical thinking on the [European] continent[, which] was dominated at the time by essentialism” (Mayr 1976: 11). As Mayr explains elsewhere,

This is the reason the great German zoologists of the first half of the nineteenth century failed so completely to solve the problem of evolution. They had been thoroughly indoctrinated in the concepts of idealistic philosophy, while the two ‘dilettantes’, Darwin and Wallace in England, had spent their time watching birds, collecting insects, and reading Malthus […], thus happily remaining unaffected by the lofty fallacies of idealistic philosophy. (Mayr 1959c: 173)

I find this to be one of the weakest and least convincing historical claims that Mayr is known for, which has been rightfully criticized and discredited by the historians who, as we noted in Sect. 2, have accused Mayr of concocting and promoting an untenable ‘Essentialism Story’ about the history of biology.^31^

One major problem with this claim that has *not* received much attention, but which I think is particularly significant, is that Mayr sets up the distinction between typological and population thinking on an unequal footing. Whereas typological thinking, or essentialism, is presented as a grand metaphysical doctrine harking all the way back to Plato, population thinking is described as a commonsensical, purely empirical standpoint utterly devoid of philosophical content. This is a mistake. Population thinking, as Mayr himself understands it, is every bit as much a philosophical thesis—with rich and far-reaching ontological and epistemological implications—as essentialism is.

Nevertheless, Mayr is quite right to point out that “just because it is so commonplace”, the uniqueness of biological individuals of population thinking “had been largely ignored by philosophers” prior to 1859 (Mayr 1982: 487). It is indeed remarkable how little attention the metaphysical proposition that every entity—let alone every *biological* entity—is different from every other has received in the history of philosophy. One interesting exception is Gottfried Wilhelm Leibniz, whose theory of monads did suggest a universe of unique individualities. His famous principle of the identity of indiscernibles likewise betrays a concern with questions of uniqueness. Leibniz even acknowledged *biological* uniqueness, writing in 1689 that “never do we find two eggs or two leaves or two blades of grass in a garden that are perfectly similar” (Leibniz 1989: 32; see also Marder 2014; Chap. 7), but these ideas were not further developed or taken up by others.

The other, and perhaps more pertinent, philosophical precedent for population thinking is the nominalist tradition that flourished during the scholastic period. The nominalists rejected essentialism and argued that only individual particulars exist. Universal kinds and other abstract categories are just names or labels produced by the human mind; they do not exist independently of us. Although Mayr (1982: 308–309) comments that “nominalism was perhaps an anticipation of population thinking”, he also questions whether restricting the ontological domain to individual particulars necessarily entailed for the nominalists of the scholastic period regarding each of them as fundamentally unique (ibid.: 862).

Be that as it may, nominalism does offer one possible way to think about the philosophical basis of population thinking, which is, after all, an anti-essentialist position. A potential worry about a nominalist interpretation of population thinking, though, is that it appears to be incompatible with a realist view of species. If only individuals exist, then all groupings and all classes, including species, are merely arbitrary, subjective constructs of the human mind. Mayr himself rejects full-blown nominalism for this reason, asserting that “[s]pecies are the product of evolution and not of the human mind” (Mayr 1969: 313). But this rejection is not well founded, as it is based on a conflation of species *taxa* with the species *category*. Nominalists will certainly want to deny the mind-independent existence of the species category, but they can still acknowledge the objective existence of species taxa, especially if they are construed as spatiotemporally bound particulars in accordance with the species-as-individuals thesis, which, as we noted in Sect. 4, is the view of species that follows most naturally from population thinking.

In one of the only explicit analyses of the metaphysics of population thinking in the literature, Nanay (2010) adopts a nominalist interpretation, but adds a twist of his own. He suggests that population thinking is not an ontological claim about entities, but about *properties*. For the population thinker, only individual property-instances—or tropes—are real. Property-types (or universals) are not. Population thinking is thus a form of *trope nominalism*. Nanay’s motivation for this interpretation is to prevent the new essentialists who have recently reconceptualized the properties that determine kind membership extrinsically as opposed to intrinsically (recall Table 1) from arguing that their historical or relational brand of essentialism is nevertheless compatible with population thinking. If population thinking denies the existence of property-types, then it rules out any version of essentialism about natural kinds.

Nanay does not just propose his trope-nominalist interpretation as *one* way of understanding population thinking, but rather proclaims it to be “*the* correct interpretation of population thinking” as Mayr conceived it, as it is the one that “does the most important theoretical work this concept is supposed to do: to sink essentialism about biological kinds” (ibid.: 92, my emphasis). This is where I think Nanay goes wrong. His concern is to argue that “evolutionary theory is really about tokens and not about types” (ibid.: 93). Consequently, he is far more interested in the contrast between particulars and universals than the contrast between individuals and populations—and he thinks Mayr is as well. But our detailed analysis of how Mayr understood his concept of population thinking dispels this presumption. Mayr’s main concern was not with particulars and universals but with individuals and populations. Population thinking, as Mayr consistently construed it (recall Table 2), is primarily a thesis about entities, not about properties.

Nanay’s approach to population thinking suffers from the same basic limitation as much of the work that we surveyed in Sect. 2, which is that it is focused on what population thinking is *not*, rather than on what population thinking *is*. Our detailed review of the literature revealed that there is a vast amount of scholarship on typology and essentialism, but hardly any at all on population thinking as a doctrine in its own right—particularly after we set aside the appropriations of the concept by Sober (1980) and Ariew (2008) that we discussed in Sect. 3. By decoupling population thinking from its familiar association with typology and essentialism, it becomes possible to consider it on its own terms, as we have started doing in this paper. One of the most salient implications of this reorientation is the realization that the foundation of population thinking must be sought not in the contrast with any doctrine that conflicts with it but in the ontological feature that the concept was coined to emphasize, namely *uniqueness*.

Unfortunately, despite the overwhelming empirical evidence for the uniqueness of biological entities—some of which we examined in Sects. 4 and 5—the very idea of uniqueness remains a blind spot in the philosophy of biology, and for that matter in biology more generally.^32^ It does not even show up in the places where one would expect it to, such as in the philosophical critiques of essentialism that we considered in Sect. 2. One refreshing exception to this general rule is the work of Wilson (1999, 2005; Wilson et al. 2007), who does specifically single out biological uniqueness, which he calls ‘intrinsic heterogeneity’, as the major cause for the failure of traditional essentialist views in biology.

Other than this, perhaps Hull’s extension of the species-as-individuals thesis to other kinds of biological entities like genes and organisms (see Hull 1978) could be interpreted as an argument for uniqueness, as it entails reconceptualizing them as spatiotemporally bound historical particulars that are evolutionarily non-recurrent and therefore unique. But this sense of uniqueness is both too broad and too narrow to capture what population thinking emphasizes. It is too broad because, strictly speaking, *every* historical process or event in the universe is non-recurrent and therefore unique in Hull’s sense. And it is too narrow because biological uniqueness is not exhausted by the diachronic non-recurrence of particular traits or lineages that is of interest to evolutionary biologists; it also involves the synchronic abundance of difference, or absence of sameness, among individuals in biological populations. Andrew Buskell and Adrian Currie have recently published a paper titled ‘Uniqueness in the Life Sciences’ (Buskell and Currie 2021), but their understanding of uniqueness is akin to Hull’s, and consequently their approach to the subject is considerably more restricted than their ostentatious title suggests.^33^

One potentially fruitful avenue to investigate biological uniqueness is to consider how it impacts the manner in which we study it. Perhaps the reason why biology has the character that it does is because it deals with entities that are ontologically unique in respects that the entities studied by the physical sciences are not—or, at the very least, that their uniqueness matters to the way we explain them. This is the central organizing theme of the theoretical biology of Walter Elsasser, a quantum theorist and geophysicist born in Germany the same year as Mayr, and who, like Mayr, emigrated to the United States in the 1930s. From the early 1950s until his death in 1991, Elsasser worked on the theoretical foundations of biology, leaving behind a large body of publications, including four monographs (Elsasser 1958, 1966, 1975, 1987) and numerous articles, many of which appeared in the *Journal of Theoretical Biology*. Regrettably, Elsasser’s work has been almost completely ignored by philosophers of biology.^34^ Even Mayr refused to engage with it in any of his published works.^35^ This is obviously not the place for a detailed examination of Elsasser’s views (for a recent reappraisal, see Gatherer 2008), but I do want to draw attention to one of his central claims, which is that the fundamental difference between physics and biology is that the former deals with *homogeneous classes*—where one member of the class is substitutable for another—while the latter has to contend with classes that are *inhomogeneous*, or, as he preferred to say in his later writings, *radically heterogeneous*.

Elsasser realized that the reason why the theoretical models of physics are so successful is that they are able to effectively treat entities of a given kind as perfectly homogeneous, abstracting away or simply ignoring any differences that may exist between them. As far as the models are concerned, every entity to which they apply can stand for any other. This interchangeability is not just possible in principle but is actually presupposed in the generalizations that the models allow us to make. This is as true for how quantum mechanics treats subatomic particles as it is for how statistical mechanics treats the molecules of an ideal gas. It is even true for how Newtonian mechanics treats bodies with the same mass, position, and momentum. In all these cases, the individuality of particulars, their *uniqueness*—to the extent that it exists at all—is theoretically of no consequence; it is irrelevant.

Contrast this with the biological situation, where the ubiquity of variation all the way up and all the way down means that we are *always* dealing with radically heterogeneous classes where it is not possible to abstract away or ignore unique differences when making generalizations. Doing so comes at a cost, as we saw in Sects. 4 and 5. Variation matters in biology; it cannot be dismissed as noise. Not only can it not be explained away, but it does much of the explaining. For one thing, it plays a key role in many biological processes, most obviously in natural selection (recall Sect. 4).

A simple way of articulating this profound difference is to remark, as Wilson et al. (2007: 192) do, that “if you’ve seen one electron (or copper molecule, or tumbler of hydrochloric acid) you’ve seen them all” as far as the relevant physical or chemical theory is concerned. On the other hand, “[i]f you’ve seen one tiger (or vertebrate or coral reef) you *haven’t* seen them all”, as individual differences in such cases are almost always relevant—and are often central—to what we are trying to understand. As we noted in Sect. 5, even in an isogenic population of cells it is *not* the case that any cell can stand in for any other.

Another pithy way of expressing Elsasser’s distinction is to state that physics is a science that deals with *generic* objects, while biology is a science that deals with *specific* objects (Montévil et al. 2016). Generic objects are all alike from a theoretical standpoint in the sense that they obey the same equations regardless of their nature. For example, Galileo’s law of free fall makes no distinction between a feather and an anvil; their physical behaviour is accounted for in exactly the same way. In biology, however, one is confronted with specific objects that invariably differ from one another in their respective properties. No two organisms are biochemically, morphologically, or behaviourally identical, which is why they are not interchangeable. The specificity of biological objects is not just a function of their formidable complexity, but also of their extreme sensitivity to their environment (recall the long list of external abiotic factors in Fig. 3), and perhaps most importantly, of their *historicity*. Biological objects are decisively shaped by the contingent historical trajectories that give rise to them (both phylogenetically and ontogenetically) in ways that purely physical objects are not.^36^

The uniqueness of biological entities helps explain many other epistemological characteristics of biology that set it apart from the physical sciences. I will just mention two often pointed out by Mayr (1982, 1988) that have been very lucidly discussed by John Beatty (1995, 1997). The first is the apparent absence of distinctively biological laws, and the second is the importance of theoretical pluralism and the abundance of what Beatty calls ‘relative significance’ controversies. It is surprisingly difficult to formulate universal generalizations about the living realm without encountering exceptions. The fact that membership in biological kinds is not determined by the possession of essential properties but is instead due to contingent evolutionary outcomes means that there is little that is truly necessary or inevitable about the biological world. Moreover, biologists, unlike physicists, do not generally expect a domain of phenomena to be fully explainable by a single theory, but instead assume that different items in the domain will require explanations in terms of different theories. Biological disputes are therefore often not about whether a particular theory is correct or not, but rather about its extent of applicability.

Mayr hoped that population thinking would help establish the autonomous status of biology and protect it from the threat of an eventual reduction to the physical sciences. Having examined the nature and implications of the ontological feature that his concept highlights—the uniqueness of biological entities—we can conclude that population thinking succeeds in doing “the most important theoretical work this concept is supposed to do”, which is *not*, as most commentators have assumed, “to sink essentialism about biological kinds” (Nanay 2010: 94), but, more fundamentally, to answer the question that Mayr chose as the title for his final book, namely *What Makes Biology Unique?* (Mayr 2004).

## Conclusions

Despite its pervasiveness and popularity, population thinking is sometimes perceived to be, in the words of Peter Godfrey-Smith (2009: 11), “a controversial and slippery idea”. This is not surprising considering what has been written about it. We have seen that although Mayr was remarkably consistent in how he characterized population thinking, the notion today is routinely confused with population-level thinking, which—though important in its own right—is a completely separate idea.

There is general agreement that population thinking is closely connected with evolutionary biology, but considerable disagreement about the basis of this association. I have suggested that population thinking is responsible for two of the most important theoretical innovations that Darwin brought to biology: the shift from a transformational to a variational model of populational change (to the extent that the transformational model can be said to deal with populations at all), and the recognition that differences between individuals are as ‘normal’ and as ‘natural’ as their similarities; the former are not deviations or accidental byproducts of the latter.

Population thinking emphasizes the primacy and ubiquity of variation in the biological domain. It tells us that we should expect to find diversity in all contexts and at every level of organization, from macromolecules to ecosystems. Intraspecific variation is not something strange or aberrant—an anomaly that needs to be mitigated or explained away. On the contrary, the default state or condition of any biological population is heterogeneity. From the perspective of population thinking, it is the generation and maintenance of sameness, or homogeneity, that is more surprising, and which cries out more loudly for explanation.^37^

Many biologists have tended to shun population thinking because the conventional assumption of homogeneity makes biological phenomena more epistemically tractable. It is much easier to study individual members of a population if we grant ourselves the license to interpret their observed differences as meaningless noise than if we are forced to acknowledge their significance. In fact, experimental biologists have traditionally gone to great lengths to ingeniously manipulate the conditions that shape biological outcomes in controlled settings to ‘reduce the noise’ so that sameness becomes expected, as this makes experimental interventions more effective, and the application and replication of results more reliable (at least in principle).

But heterogeneity is not only inescapable as an empirical feature of the living world; it is also almost always theoretically meaningful. This crucial realization is seeping into every area of biology. We have looked particularly closely at the current situation in molecular biology, as the rise of population thinking there has been quite unexpected and might ultimately turn out to be especially consequential. As more molecular biologists adopt techniques that allow them to study not just individual cells but also individual molecules inside individual cells (as opposed to relying on population-averaging methods), it might eventually become necessary to rethink our understanding of even the most basic cellular processes.

I hope that this paper helps to draw our attention away from the tired question of what population thinking is a reaction against or is supposed to have historically overthrown—which has been the main focus of almost all scholarship on this topic—and toward the question of what it is actually about: the uniqueness of biological entities. Uniqueness has remained a largely unexplored notion in the philosophy of biology, perhaps because recognizing it has been perceived to be incompatible with theorizing (‘if everything really is unique, then general theories will always fail!’). But this is wrong. Population thinking does not prevent us from theorizing; it simply requires that our theories factor in the uniqueness it emphasizes. We have seen that Elsasser’s ideas—long overlooked by philosophers of biology—are a prime example of just what such an approach to biological theory might look like.

One key implication of population thinking is that it seems to put the finger on why biology is, in so many interesting respects, different from the physical sciences. I have only been able to explore this intriguing idea in a preliminary way. Much more remains to be said about it, as well as about the nature and sources of biological uniqueness (I have distinguished three sources—complexity, context-sensitivity, and historicity—but they each need to be examined in greater detail). And, of course, there is the question of how the physical world of homogeneous entities gave rise to the biological world of heterogeneous entities.

Population thinking is a rich philosophical thesis, with far-reaching implications that we have just begun to carefully consider. It seems only fair that I let Mayr have the last word: “Nothing is perhaps more characteristic of the world of living organisms than the universality of uniqueness. [...] We will not have an acceptable philosophy of science until all the consequences of the uniqueness principle in the living world [i.e., *population thinking*] are properly accommodated by such a philosophy” (Mayr 1985: 55–56).

## Data Availability

No datasets were generated or analysed during the current study.

## Footnotes

1 This abridged 1976 version has in turn been reprinted many times, including in all three editions of Elliott Sober’s popular anthology *Conceptual Issues in Evolutionary Biology* (Sober 1984a, 1994, 2006).

2 David Hull—another protégé of Mayr—similarly stated that the advent of population thinking is, “[t]o my way of thinking, *the* central conceptual change in recent biology” (Hull 1994: 382).

3 In fact, at the start of their acclaimed *Not By Genes Alone*, Peter Richerson and Robert Boyd declare that “[p]opulation thinking is the core of the theory of culture we defend in this book” (Richerson and Boyd 2005: 5).

4 The irony here is that without the typological belief in the objective relatedness of some species with others as reflecting the same ‘Unity of Type’, there would have been no grounds to hypothesize common ancestors for particular groups of species.

5 Another close contender is Philip Henry Gosse, but his views, like Agassiz’s, had little discernible influence (Wilkins 2009: 118–119).

6 As Winsor (2006) has detailed, Mayr started using ‘essentialism’ pejoratively as a synonym for ‘typology’ and ‘typological thinking’ in the late 1960s (e.g., Mayr 1968). This was partly a result of reading a paper by Hull with the dazzling title ‘The Effect of Essentialism on Taxonomy—Two Thousand Years of Stasis’ (Hull 1965), which Hull had written for a graduate seminar taught by Karl Popper, who had coined the term ‘essentialism’ two decades earlier (Popper 1945).

7 This remains a common way of understanding what variation is. For example, the *Oxford English Dictionary* defines biological variation as: ‘Deviation or divergence in the structure, character, or function of an organism from those typical of or usual in the species’.

8 As Sober (1984b: 168) later intimated, “hypotheses of group selection are examples of population thinking *par excellence*”.

9 Hey (2011: 255–256) provides many earlier examples of this confusion in his own paper.

10 In *Natural Inheritance*, Galton amusingly writes that “[i]t is difficult to understand why statisticians commonly limit their inquiries to Averages, and do not revel in more comprehensive views. Their souls seem as dull to the charm of variety as that of the native of one of our flat English counties, whose retrospect of Switzerland was that, if its mountains could be thrown into its lakes, two nuisances would be got rid of at once” (Galton 1889: 62).

11 Unsurprisingly, Fisher is the main target of Mayr’s (1959b) famous critique of ‘bean bag genetics’, which Mayr criticizes, among other things, for neglecting the role of the individual in evolution.

12 Notice how similar Sober’s characterization of population thinking is to the ones in Table 3—and how different it is from the ones in Table 2.

13 Sober’s classic paper on population thinking, but not Mayr’s, was also reprinted in the anthology *The Units of Evolution: Essays on the Nature of Species* (Ereshefsky 1992).

14 In Walsh’s own words, “the basic unit of organisation should not be seen as the population but the individual. Individual thinking—not population thinking—is crucial to any understanding of adaptation” (Walsh 2000: 151). More recently, Walsh has taken issue with what he dubs ‘The Paradox of Population Thinking’ (which, again, actually concerns population-level thinking) in ways that have further entangled matters (Walsh 2019).

15 Considering how often Mayr wrote about population thinking in his later years (recall Table 2), it is surprising that he did not discuss Sober’s interpretation of his notion in any of his publications. In private correspondence, however, Mayr objected to Sober’s claim that individual organisms are *less* real for the population thinker than they are for the essentialist, adding that on Sober’s interpretation it is impossible to understand how natural selection works (Mayr to Sober, November 7, 1978 [HUGFP 74.7 Box 26, Folder 1299]; Sober to Mayr, June 18, 1979 [HUGFP 74.7 Box 28, Folder 1314], Papers of Ernst Mayr, Harvard University Archives). Two decades later, Chung reported in his unpublished dissertation that Mayr personally communicated to him that “Sober’s account [of population thinking], while philosophically important, does not accord with [his] views very well” (Chung 2000: 20).

16 For example, what Ariew (2008) calls population thinking closely corresponds to what Erwin Schrödinger referred to as ‘order-from-disorder’ reasoning in *What is Life?* (Schrödinger 1944), or what Walsh (2019) has more recently termed ‘higher-order effect explanations’. Ariew himself has recently suggested that in order to avoid terminological confusions we should rename Sober’s (1980) conception of population thinking—which I have called here ‘population-level thinking’—as ‘statistical thinking’ (see Ariew 2022).

17 This distinction was proposed by Richard Lewontin, who discusses it in several places (Lewontin 1983: 65–66, 2000: 8–9, 2001: 53–54, 66–67). Mayr occasionally mentions it as well, crediting Lewontin, in his late writings (e.g., Mayr 1988: 15–16, 457–458).

18 Stephen Jay Gould devoted an entire book (i.e., Gould 1996) to explaining how the Darwinian emphasis on the primacy of variation forces us to relinquish the cherished notion of evolutionary progress (assumed in transformational theories).

19 This innovation is partly addressed in Sober’s (1980) lucid examination of how the Aristotelian natural state model was undermined by Darwin’s theory, but Lewontin, again, discussed it earlier and more explicitly (Lewontin 1974, 2001).

20 Lewontin (1974) interestingly suggests that Gregor Mendel’s pioneering work (and the subsequent advent of genetics) also helped bridge the ontological gap between similarity and difference by empirically demonstrating that heredity and variation are concomitant outcomes of the same causal processes. Due to space limitations, I will not explore this idea here.

21 It is precisely to avoid this confusion and to highlight the distinctive heterogeneity of *biological* populations that Mayr, in his later years, preferred to use the term ‘biopopulation’, instead of just ‘population’, in his work (e.g., Mayr 1988, 2001, 2004).

22 Hey 2011 is the only discussion of population thinking I have encountered that explicitly recognizes this.

38 Indeed, Chung (2000, 2003) goes as far as to argue that population thinking enabled Mayr to demarcate (and thereby vindicate) the naturalist tradition that gave rise to evolutionary biology from the experimental tradition embodied in the flashier, newer molecular biology.

23 For a detailed examination of the problematic appeal to engineering metaphors in molecular biology, see Nicholson 2020.

24 It should not escape the reader’s notice that the contrast between graded and stochastic models of gene expression in Fig. 4 closely resembles the contrast between transformational and variational models of populational change in Fig. 2. This is not a coincidence. The former can be regarded as an instantiation of the latter.

25 The same can be said for the directed movement of any motor protein transporting cargo inside the cell.

26 It has been estimated that in mammals at least 25% of all proteins are intrinsically disordered (Dunker et al. 2008).

27 It is worth remembering, though, that reports of non-genetic heterogeneity are not new (e.g., Spudich and Koshland 1976). However, only in recent years has the phenomenon become widely documented and generally accepted.

28 “The crucial aspect of the population concept to have emerged in evolutionary biology”, Mayr writes in reference to population thinking, “is the *genetic uniqueness* of the composing individuals” (Mayr 1997: 211, my emphasis).

29 In recent work, I have suggested that a major reason for this is historical—having to do with the particular physical understanding of cellular order that molecular biology inherited from Schrödinger’s *What is Life?* (Nicholson in press).

30 The breeders’ method of artificial selection was also instrumental in helping Darwin articulate his principle of natural selection. No wonder, then, that Darwin chose to start the *Origin* with a chapter on the practices of animal and plant breeders, and that he later went on to write a massive, two-volume work on this topic (Darwin 1868).

31 Still, no historian has lampooned this aspect of the Essentialism Story as brazenly as the evolutionary biologist Günter Wagner, who in a section of his essay review of Amundson 2005 titled ‘Was everyone before Darwin really an idiot?’ writes that Mayr’s “cartoon version of the history of biology has it that there was the dark age in which the greatest minds of western culture were held hostage to the vile philosophical ideas of idealism, or what ever it was called at the time. The stranglehold of these philosophical preconceptions where [sic] keeping biological research confined in the unenlightened dungeon of typology and species fixism. And then, out of the blue sky of Southern England, comes the genius that set biology free by breaking the shackles of idealism and replacing them with the wings of population thinking” (Wagner 2007: 151).

32 Even the related but more familiar notion of *variation* has not received much attention as a theoretical concept in its own right—either in biology or in philosophy. As Benedikt Hallgrímsson and Brian Hall observe, “[a]lthough implicit in virtually all research in the biological sciences, whether one is seeking understanding at the genetic, developmental, organismal, species, population, or ecologic/community levels, variation is seldom treated as a subject in and of itself” (Hallgrímsson and Hall 2005: 1).

33 The only other recent engagement with biological uniqueness that I am aware of comes from ethnographic studies conducted by philosophers involved in a large interdisciplinary collaboration with behavioural biologists, ecologists, and evolutionary biologists interested in the nature of biological individuality (see Kaiser and Trappes 2021; Trappes 2022).

34 Wilson is, again, a refreshing exception here (see Wilson 2005, 2007; Wilson et al. 2007). Although he does not discuss Elsasser’s work in any detail, he does appear to have derived his conviction of the intrinsic heterogeneity of the living world from his reading of Elsasser.

35 In a letter to Conrad Hal Waddington, Mayr remarks that “I find Elsasser quite sterile even though he has written much on biology” (Mayr to Waddington, January 17, 1966 [HUGFP 74.7 Box 14, Folder 919], Papers of Ernst Mayr, Harvard University Archives).

36 Of course, this is not to say that history never matters to physical objects. Stars, for instance, undergo an intricate sequence of transformations that determine their behaviour, and the same is true at much shorter timescales for self-organizing dissipative structures like tornadoes. The difference is that these historical trajectories are fully pre-defined by the relevant theory, whereas the historical trajectories of biological objects are largely unpredictable and unrepeatable—they are *unique*.

37 I cannot resist making the observation that some of the consequences of embracing population thinking bear a striking resemblance to those of adopting a processual viewpoint in biology (see Dupré and Nicholson 2018). Very briefly and schematically: processualism advocates a shift from taking stability for granted as the default state and trying to understand why things change, to taking change for granted as the default state and trying to understand why things are—or appear—stable. Analogously, population thinking advocates a shift from taking sameness for granted as the default state and trying to understand why things differ, to taking difference for granted as the default state and trying to understand why things are—or appear—the same (perhaps one could even imagine as a third possibility a sort of parity thesis that confers equal ontological weight to sameness and difference). In any event, a closer examination of the relation between processualism and population thinking will need to be left for another occasion.
